# Stabilizing the Hexacyanotrimethylenecyclopropane Electron Acceptor—Structural and Photophysical Characterization

**DOI:** 10.1002/anie.5081033

**Published:** 2026-05-25

**Authors:** Jan P. Soyka, Alok Mahata, Anja Wiesner, Jennifer Hartfiel, Christian E. Halbig, Katharina J. Franke, Ute Resch‐Genger, Biprajit Sarkar, Siegfried Eigler

**Affiliations:** ^1^ Institute of Chemistry and Biochemistry Freie Universität Berlin Berlin Germany; ^2^ Fachbereich Physik and Halle‐Berlin‐Regensburg Cluster of Excellence CCE Freie Universität Berlin Berlin Germany; ^3^ Bundesanstalt Für Materialforschung und –prüfung (BAM) Department 1 Division Biophotonics Berlin Germany

**Keywords:** [3]radialene, crystal structure, electron acceptor, fluorescence, organic oxidant

## Abstract

Hexacyanotrimethylenecyclopropane (**CN6CP**) is an exceptionally strong organic electron acceptor in its neutral form, and widely applied for molecular doping to induce charge transfer processes and enable electrochemical systems. Yet, its fundamental molecular properties have remained largely unknown. Here, we show the first comprehensive structure‐analytical characterization of **CN6CP**, enabled by an improved, low‐temperature synthesis and the first solid‐state structure of the neutral compound. The resulting procedure affords isolable, crystalline **CN6CP** that is stable for weeks at –30°C and can be recrystallised. Across all redox states, combined IR/Raman, UV–Vis and NMR measurements, together with NICS calculations, reveal an oxidation‐state‐dependent redistribution of electron density. These data show that **CN6CP** possesses a σ‐aromatic cyclopropane core with tunable π‐delocalisation, which is enhanced upon reduction while the additional charge is predominantly localised on the exocyclic acceptor framework. Cyclic voltammetry experiments unveil two reversible one‐electron processes and an exceptionally low LUMO energy of –5.85 eV, which is the lowest reported for small organic molecules being significantly lower than those of benchmark acceptors such as F_4_TCNQ or F_6_TCNNQ. All together, these findings establish **CN6CP** as a structurally unique, extremely strong electron acceptor and provide the molecular basis underlying its performance in organic electronics and redox‐active materials.

## Graphical abstract

An improved low‐temperature synthesis enables the first comprehensive structural and spectroscopic characterisation of neutral hexacyanotrimethylenecyclopropane (**CN6CP**). Combined IR/Raman, UV–Vis and ssNMR analyses reveal a deeper understanding of its oxidation‐state‐dependent charge redistribution, σ‐aromaticity of the cyclopropane core, and redox‐tuneable electronic properties.

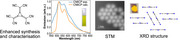

## 1 Introduction

**FIGURE 1:**
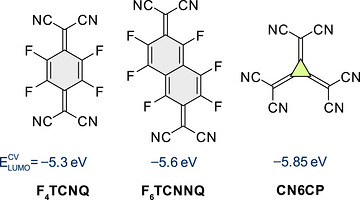
Chemical structures of selected benchmark cyano‐based electron acceptors and **CN6CP** together with their LUMO energy levels relative to vacuum. The LUMO values were estimated from cyclic voltammetry data referenced to Fc/Fc^+^ and compiled from the literature and this work [4, 12, 13].

Organic electron acceptors with high electron affinity, such as tetracyanoquinodimethane (TCNQ) and their fluorinated derivatives tetrafluorotetracyanoquinodimethane (F_4_TCNQ) and hexafluorotetracyanonaphthoquinodimethane (F_6_TCNNQ), play a key role in organic electronics and charge transfer materials [1, 2, 3, 4]. Their low‐lying lowest unoccupied molecular orbital (LUMO) levels make them strong oxidants and enable controlled electron transfer [5, 6, 7, 8, 9]. While these molecules are well studied, hexacyanotrimethylenecyclopropane (**CN6CP**), first reported by Fukunaga in 1976 [10, 11], embodies one of the strongest small‐molecule organic electron acceptors known (see Figure 1), which is, however, structurally relatively unexplored, even though potential applications become more and more relevant.

Hexacyanotrimethylenecyclopropane (**CN6CP**) has received renewed attention in the last decade following an improved synthesis by Kiriy et al. [14], thereby enabling its use as a strong electron p‐dopant [15, 16], e.g. in organic semiconductors [17, 18, 19, 20, 21], including organic field‐effect transistors (OFETs) [22, 23], and large‐bandgap organic light‐emitting diodes (OLEDs) [17, 24]. In addition, **CN6CP** forms charge‐transfer complexes with electron‐rich donors [25, 26], which enable molecular magnetism [27, 28], and is being used increasingly in organic thermoelectrics [29, 30, 31, 32, 33, 34, 35, 36]. However, most of these applications rely on the in situ formation of the air‐ and water‐stable radical anion **CN6CP^∙−^** [16, 22, 31, 37]. We have recently employed the neutral structure as strong acceptor [38] and, in a slightly modified form as an electron‐withdrawing moiety in push‐pull fluorescence dyes [39, 40]. The radical anion itself can also be directly used, e.g., as a redox‐active component in catholyte batteries and other electrochemical systems [31, 37]. Despite its growing relevance, there is still a lack of fundamental molecular characterisation for neutral **CN6CP**. So far, neither spectroscopic details nor information on the solid‐state structure of the neutral molecule is available.

Here, we present an improved synthesis of neutral **CN6CP**, also leading to a stable solution and the first singlecrystal X‐ray diffraction structure as well as comprehensive spectroscopic analysis of both the neutral compound and its radical anion. We also show that the neutral molecule is sublimable and forms self‐assembled structures on a metal surface.

## 2 Results and Discussion

**FIGURE 2:**
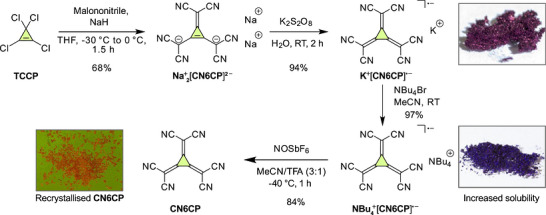
Modified synthesis of **CN6CP** starting from tetrachlorocyclopropene (TCCP) yielding **Na** ^**+**^_**2**_[**CN6CP** **]** ^**2–**^ by nucleophilic substitution with malononitrile. Subsequent oxidation yields **K** ^**+**^ **[CN6CP]** ^**−**^. Counterion exchange yields the more soluble radical anion salt **NBu**_**4**_^**+**^ **[CN6CP]** ^**−**^, which can be oxidised at low temperature with, for example, NOSbF_6_ to give **CN6CP**. Insets: Photographs of compounds **K** ^**+**^ **[CN6CP]** ^**−**^, **NBu**_**4**_^**+**^ **[CN6CP]** ^**−**^, and of **CN6CP** crystallised from a solution of MeCN/TFA with small amounts of NOSbF_6_.

The previously reported synthesis employed the poorly soluble radical anion **K^+^[CN6CP^∙−^]** in MeCN/TFA or MeNO_2_. [14] The radical becomes well soluble by exchanging K^+^ to tetra‐*n*‐butylammonium forming **NBu_4_^+^[CN6CP^∙−^]** enabling the controlled oxidation to **CN6CP** at low temperatures of –40°C by the dropwise addition of a suitable oxidant, such as NOSbF_6_, NOPF_6_ or NO_2_SbF_6_ in MeCN/TFA or MeNO_2_. In the course of the reaction, orange crystals are formed (see Figure 2), which can be collected by filtration and stored under argon at –30°C for several weeks. However, over time, the compound darkens and develops a metallic shine, indicating the formation of the stable radical anion. We note that a solution of compound **CN6CP** in MeCN/TFA (4:1) containing small amounts of NOSbF_6_ remains stable for at least two months at temperatures below –30°C (for further details see Figure S4) and can even be recrystallis‐ed from the refluxing solution. Further purification of compound **CN6CP** is possible under reduced pressure since only the neutral form can be sublimed.

**FIGURE 3:**
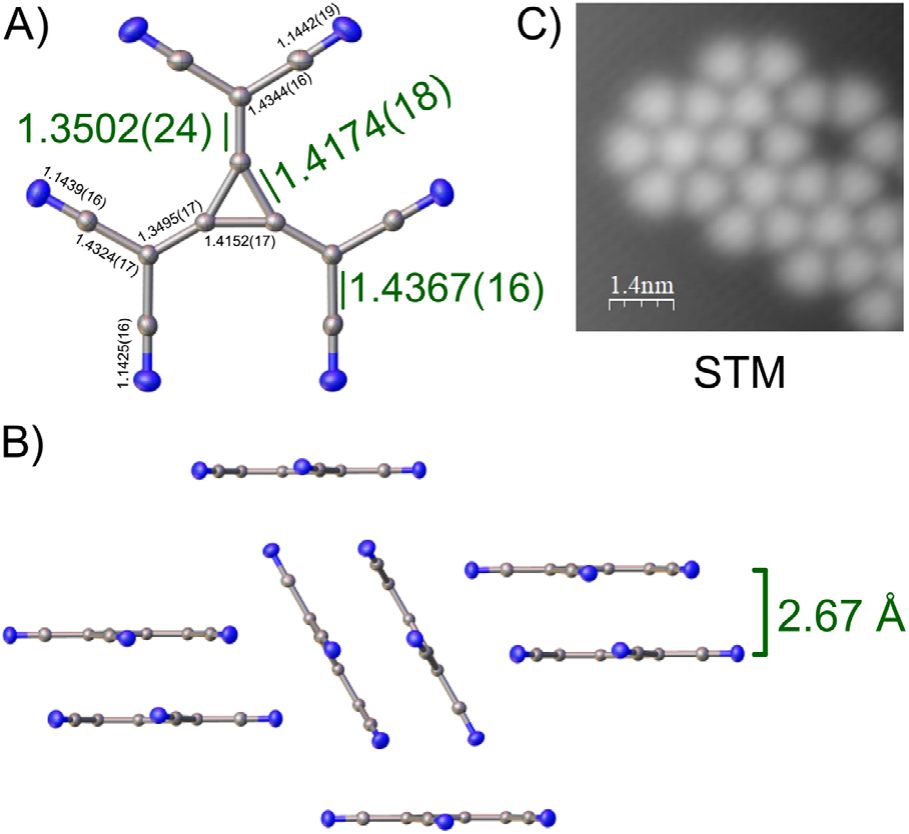
(A) Solid‐state structure of compound **CN6CP** determined by single‐crystal X‐ray diffraction viewed form the top. **CN6CP** is essentially planar, with small deviations from coplanarity in the range of 1–2° and exhibits a C_2_ symmetry axis. Symmetry‐equivalent bond lengths are omitted. (B) The 3D packing of **CN6CP**. The molecule crystallises with one unit of MeCN, omitted for reasons of clarity. Thermal ellipsoids are shown at 50% probability level. Carbon is displayed in grey and nitrogen in blue [41]. (C) Scanning tunnelling microscopy image of the 2D packing of compound **CN6CP** on Cu(111) displays a honeycomb structure. (Scanning parameters *I*  =  50 pA, *V*  =  1.0 V).

Next, we investigated the solid‐state structure of compound **CN6CP**, which could be confirmed by single‐crystal X‐ray diffraction (Figure 3). [41]. Data were collected at 167(2) K using Mo*K_α_* radiation. The neutral form of compound **CN6CP** crystallises with one equivalent of MeCN solvate in the orthorhombic space group *Pnma* with *Z* = 4 and *Z*′ = 0.5. The unit cell parameters are *a* = 9.4267(12) Å, *b* = 11.8644(13) Å, *c* = 12.0518(16) Å, and *V* = 1347.9(3) Å^3^ with α = β = γ = 90° [41]. **CN6CP** crystallises in a sandwich (double) herringbone type structure with a molecular distance of approximately 2.67 Å. Multiple short intermolecular N to C contacts between neighbouring nitrile groups are observed in the crystal packing, consistent with weak dipolar nitrile–nitrile interactions (see Figures S12 and S21) [42]. The crystallographic C2 symmetry leads to a slight differentiation of formally symmetry‐equivalent C–C bonds. However, the deviations are within experimental uncertainty and correspond to only a very minor distortion from ideal C3 symmetry. The molecule is essentially planar with all torsion angles below 1.8°. The C–C bond length in the cyclopropane ranges from 1.4174(18) Å to 1.4152(17) Å. The exocyclic C–C bonds from the ring to the dicyanomethylene range from 1.3495(17) Å to 1.3502(24) Å. These bonds are significantly shorter compared to those of the previously reported radical anion [43], with bond lengths of 1.4002(24) Å–1.3998(22) Å for the cyclopropane and longer exocyclic bonds (1.3708(24) Å–1.3747(21) Å) (see Figure S20 and 21) [43]. Likewise, several crystal structures of a dianionic **CN6CP** salt are reported [37, 44]. For example, in the **(NBu_4_^+^)_2_[CN6CP]^2–^** salt, the ring C–C bonds are between 1.385(5) and 1.393(7) Å, whereas the corresponding exocyclic bonds range from 1.386(7) to 1.407(5) Å. In addition, the bond length of the central carbon of the dicyanomethylene group to the nitrile carbon is longer compared to the dianion (1.411(9) Å–1.421(8) Å), ranging from 1.4323(17) Å to 1.4367(16) Å in neutral **CN6CP**. Despite the small bond‐length variations within each structure, the crystallographic data consistently show a progressive shortening of the ring C–C bonds and a concomitant lengthening of the exocyclic bonds upon reduction from the neutral species to the radical anion and further to the dianion. These structural changes suggest that the additional electron of the radical anion is at least partially localised within the cyclopropane ring and the central carbon of the dicyanomethylene (Figure S7).

The solid‐state structure was further investigated after sublimation under ultra‐high vacuum conditions on an atomically clean copper surface. Scanning tunnelling microscope (STM) images reveal that **CN6CP** adsorbs in a flat configuration on Cu(111) with the individual molecules exhibiting a triangular shape as expected from their structure (Figure 3C). The close‐up view suggests several honeycomb motifs with the centre often being filled with an additional **CN6CP** molecule (for larger‐scale images, see Figure S17). These arrangements strongly differ from the bulk crystal structure and imply that electron‐rich CN groups are facing each other. Such situations are typically stabilised by adatoms from the underlying metal surface [45, 46, 47]. These STM data primarily demonstrate that neutral **CN6CP** can be sublimed under ultra‐high vacuum conditions and deposited intact on Cu(111), where it forms an ordered, substrate‐templated assembly.

Over all three oxidation states, the Raman and infrared vibrational modes of the cyclopropane unit and the nitrile groups exhibit a characteristic trend for the respective oxidation state. In the FT‐IR spectra, the cyclopropane vibration shifts from 1564 cm^−1^ of neutral **CN6CP** to 1485 cm^−1^ in radical anion **CN6CP^∙−^** and 1428 cm^−1^ in dianion **CN6CP^2–^**, while the nitrile stretching vibration changes little from 2219 cm^−1^ (**CN6CP**) to 2213 cm^−1^ (**CN6CP^∙−^**) and splits into two signals (2206/2173 cm^−1^) in **CN6CP^2–^**. Raman spectra show the corresponding $\tilde{\nu }$(CN) bands at 2227 (**CN6CP**), 2218 (**CN6CP^∙−^**), and 2299/2257 cm^−1^ (**CN6CP^2–^**) together with systematic shifts of the symmetric and asymmetric cyclopropane modes from 1762 (${\tilde{\nu }}_{s}$, **CN6CP**) to 1848 (${\tilde{\nu }}_{s}$) and 1471(${\tilde{\nu }}_{as}$) for **CN6CP^∙−^** to 1932 (${\tilde{\nu }}_{s}$) and 1556 cm^−1^ (${\tilde{\nu }}_{as}$) for **CN6CP^2–^**. Taken together, the IR and Raman trends clearly demonstrate the progressive strengthening and redistribution of electron density within the acceptor core upon single and double reduction. The population analysis Δ*ρ* (Figure S8) shows that upon reduction electron density is removed from the σ‐framework whereas the out‐of‐plane π‐orbitals become more populated across the acceptor core unit. This σ‐ to π‐redistribution decreases the force constants of the IR‐active asymmetric modes but enhances the polarisability and stiffness of the Raman‐active symmetric modes. Consequently, IR bands shift to lower and Raman bands to higher wavenumbers along the reduction series.

**FIGURE 4:**
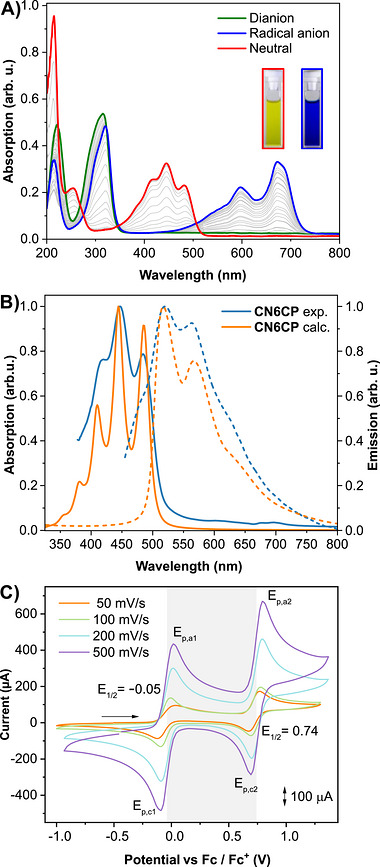
(A) Changes in the UV/Vis/NIR spectra of **CN6CP** in MeCN with 0.1 m Bu_4_NPF_6_ during the first and second oxidation with an Au working electrode. The UV/Vis of the dianion is depicted in green, radical anion in Red and neutral in Blue. (B) Experimental (blue) and calculated (orange) absorption (solid line) and emission (dotted line) spectra of **CN6CP**. Calculated at the *ω*B97D3 level with the def2‐TZVPD basis set and the conductor‐like polarizable continuum model (CPCM) for MeCN using the ESD simulation. The calculated spectra were aligned with experimental data with respect to the band maxima. (C) Cyclic voltammograms of a 0.1 mm solution of **CN6CP** with 0.1 m NBu_4_PF_6_ in MeCN at different scan rates.

As shown in Figure 4, the oxidation state has a significant impact on the UV–Vis spectra. For compound **CN6CP^2–^** two absorption bands in the UV region at 220 and 315 nm are observed. For the radical anion (**CN6CP^∙−^**) two broad bands are in the visible region at 597 and 673 nm resulting is a deep blue colour in solution. Two additional absorption bands are in the UV at 214 and 320 nm. The neutral compound **CN6CP** exhibits broad absorption bands with three maxima in the visible at 416, 445, and 482 nm and one strong absorption in the UV at 215 nm. If dissolved in a suitable solvent, such as MeCN, the compound **CN6CP** forms a yellow‐green solution, with a moderate emission and a fluorescence quantum yield of 5% in MeNO_2_/TFA. The emission spectra of **CN6CP** displays a vibronic fine structure with two maxima at 447 and 485 nm. The vibronic fine structure observed in the absorption (and emission) spectrum of **CN6CP** originates from coupling of the electronic S_0_–S_1_ (respectively S_1_–S_0_) transition to distinct vibrational modes of the central cyclopropane core. Quantum chemical simulations reproduce the experimentally observed band spacing and relative intensities well, indicating that the vibrational motions of the three‐membered ring dominate the vibronic splitting (Figures S14 and S15). These vibrational modes contribute significantly to the spectral broadening and define the characteristic vibronic progression in both absorption and emission.

Cyclic voltammetry of **CN6CP** reveals two reversible one‐electron oxidation processes (see Figure 4B). The half‐wave potentials for the first and second oxidation are –0.05 V and 0.74 V vs. Fc/Fc^+^, respectively. Based on these values, a LUMO energy level of –5.85 eV for the neutral species and a SOMO‐derived energy level of –5.05 eV for the radical anion were calculated (see Equation S1, for details of the conversion and energy calibration).

In comparison, widely used electron‐withdrawing acceptors such as F_4_TCNQ and F_6_TCNNQ exhibit higher LUMO energies of approximately –5.3 and –5.6 eV [48], respectively. Relative to its constitutional isomer hexacyanobenzene, which features a LUMO energy of approximately –5.04 with –0.05 eV versus Fc/Fc^+^ [49], **CN6CP** displays a stronger electron‐accepting ability despite sharing the same molecular formula and functional groups. This pronounced stabilisation highlights the unique electronic properties imparted by the cyclopropane core bearing three dicyanomethylene substituents. To the best of our knowledge, the resulting LUMO energy level represents the lowest reported for a small organic molecule.

The three‐membered ring exhibits pronounced magnetic shielding in all redox states, characteristic of cyclopropane‐type σ‐aromaticity [50, 51]. As the neutral **CN6CP** neither dissolved adequately nor remained stable in solution, its ^13^C NMR spectrum was acquired in the solid state. The experimental ^13^C NMR shifts of the CP‐ring carbons are consistent with this picture: the neutral species **CN6CP** shows a signal at 134 ppm (solid state), while the dianion **CN6CP^2–^** displays a substantially more shielded resonance at 124 ppm in DMSO‐*d*_6_. Although these values were obtained under different conditions and are therefore not directly quantitatively comparable, they are in line with increased shielding upon reduction. Increasing electron count enhances π‐electron delocalisation across the radialene framework (Figure S9), yet the calculated NICS profiles indicate that classical Hückel π‐aromaticity is not fully developed. Instead, the system displays a mixed aromatic character, wherein σ‐aromaticity dominates but is augmented by increasing π‐contributions upon reduction.

Both, NICS(0) values (neutral: –20.2 ppm; radical anion: –27.4 ppm; dianion: –31.3 ppm) and NICS(1)_zz_ values (neutral: –13.1 ppm; radical anion: –15.6 ppm; dianion: –17.8 ppm) become progressively more negative, consistent with strengthening σ‐aromaticity as well as modest, redox‐dependent π‐ring current contributions, in agreement with the calculated bonding analysis and the observed crystallographic trends. The additional electron density localises mainly on the central carbon atoms of the dicyanomethylene groups, as reflected by the ^13^C NMR shift from 84.8 ppm (**CN6CP**) to 24.9 ppm (**CN6CP^2–^**) and confirmed by MBIS (Minimal Basis Iterative Stockholder, a variant of the Hirshfeld method) [52] charge population analysis (Figure S10). The same overall trend is reflected in the Harmonic Oscillator Model of Aromaticity (HOMA) (Table S9).

Overall, neutral species **CN6CP** bearing a cyclopropane ring with three exocyclic double bonds is well‐described as a [3]radialene. At the same time, the elongated exocyclic bonds and their bond orders of only about 1.6 show that this description is somewhat idealised. Upon reduction, the bonding pattern changes progressively. In the dianion **CN6CP^2–^** the exocyclic bonds show only weak double‐bond character, with bond orders of about 1.2, similar to those of the shortened bonds within the cyclopropane core (ca. 1.2), while the radical anion **CN6CP^∙−^** adopts an intermediate bonding situation between the neutral species and the dianion (Table S5). Both reduced forms are therefore best described as retaining dominant σ‐aromatic character while showing increasing π participation upon reduction, even though the additional negative charge resides primarily on the dicyanomethylene substituents (Figure S8–S10).

## 3 Conclusion

We report a substantially improved and robust synthesis of **CN6CP** that overcomes previous solubility and stability limitations. The enhanced synthetic route enables the reliable isolation of crystalline **CN6CP** with a significantly improved low‐temperature shelf stability, allowing a complete structure‐analytical and spectroscopic characterization. Further, the first solid‐state structures of the neutral molecule are reported determined in single crystals and on surfaces. The comprehensive spectroscopic characterization of the three relevant redox states reveals signatures representative for the oxidation states. Structural, vibrational, and electron‐density analyses reveal a pronounced charge redistribution upon reduction, consistent with the strong acceptor character of the cyclopropane–dicyanomethylene framework. Cyclic voltammetry shows two fully reversible one‐electron processes, unveiling an exceptionally low LUMO energy of –5.85 eV‐significantly below those of established acceptors such as F_4_TCNQ and F_6_TCNNQ. The distinct optical and electronic signatures of the neutral, radical anion, and dianion species highlight the unique redox behaviour of **CN6CP**, which combines a σ‐aromatic cyclopropane core with increasing π‐delocalisation upon reduction. The combination of aromaticity and a low‐lying LUMO level makes **CN6CP** one of the strongest known small‐molecule electron acceptors and provides a robust foundation for its targeted application in organic electronics and charge‐transfer materials.

## Author Contributions

**Jan P. Soyka**: writing – original draft, conceptualization, visualization, writing – review and editing, formal analysis, data curation, investigation, methodology, validation. **Alok Mahata**: methodology, data curation, validation. **Anja Wiesner**: methodology, formal analysis, data curation, validation. **Jennifer Hartfiel**: methodology. **Christian E. Halbig**: methodology. **Katharina J. Franke**: methodology, funding acquisition, resources. **Ute Resch‐Genger**: methodology, funding acquisition, resources, data curation. **Biprajit Sarkar**: methodology. **Siegfried Eigler**: conceptualization, resources, funding acquisition, writing – review and editing, project administration.

## Conflicts of Interest

The authors declare no conflict of interest.

## Supporting information




**Supporting File 1**: Additional information and findings of this study are available in the Supporting Information of this article. The authors have cited additional references within the Supporting Information [54, 55, 56, 57, 58, 59, 60, 61, 62, 63, 64, 65, 66, 67, 68, 69, 70, 71, 72, 73, 74, 75, 76, 77].

**Supporting File 2**: anie72648‐sup‐0002‐SuppMat.pdf.

## Data Availability

The data that support the findings of this study are openly available in Refubium at https://doi.org/10.17169/refubium‐50747 [53].
