# Supplementary material for: Stabilizing the Hexacyanotrimethylenecyclopropane Electron Acceptor—Structural and Photophysical Characterization
Source: Angew Chem Int Ed Engl. 2026 May 25;65(30):e5081033. doi: 10.1002/anie.5081033 (PMC13383071; doi:10.1002/anie.5081033)
Supplement: Supplementary file 2 — Supporting File 2: anie72648‐sup‐0002‐SuppMat.pdf. [file ANIE-65-e5081033-s002.pdf]

Supporting Information

Stabilizing the Hexacyanotrimethylenecyclopropane Electron Acceptor – Structural and Photophysical Characterization

Jan P. Soyka,[a] Alok Mahata,[b] Anja Wiesner,[c] Jennifer Hartfiel,[d] Christian E. Halbig,[a] Katharina J. Franke,[d] Ute Resch-Genger,[e] Biprajit Sarkar[b] and Siegfried Eigler*[a]

a. *M. Sc. J. Soyka and Prof. Dr. S. Eigler*
Institute of Chemistry and Biochemistry
Freie Universität Berlin
Altensteinstraße 23a, 14195 Berlin, Germany
E-mail: siegfried.eigler@fu-berlin.de

b. *M. Sc. A. Mahata, Prof. Dr. B. Sarkar*
Institute of Chemistry and Biochemistry
Freie Universität Berlin
Fabeckstraße 34–36, 14195 Berlin Germany

c. *Dr. A. Wiesner*
Institute of Chemistry and Biochemistry
Freie Universität Berlin
Fabeckstr. 34/36, 14195 Berlin

d. *M. Sc. J. Hartfiel, Prof. Dr. K. J. Franke,*
Fachbereich Physik and Halle-Berlin-Regensburg Cluster of Excellence CCE
Freie Universität Berlin
Arnimallee 14, 14195 Berlin, Germany

e. *Dr. U. Resch-Genger*
Bundesanstalt für Materialforschung und –prüfung (BAM),
Department 1, Division Biophotonics
Richard-Willstätter Straße 11, 12489 Berlin, Germany.

Table of Contents

General Information 2

Disodium 2,2',2''-(cyclopropane-1,2,3-triylidene)trimalononitrile 5

Potassium 2,2',2''-(cyclopropane-1,2,3-triylidene)trimalononitrile anion radical 6

Tetrabutylammonium 2,2',2''-(cyclopropane-1,2,3-triylidene)trimalononitrile anion radical ... 7

Spectroscopic Measurements 9

Cyclic voltammetry 10

Thermogravimetric analysis (TGA) 13

Computational Details 14

Electron distribution 16

Charge distribution 18

Bond Order calculations 20

Non-bonding orbitals (NBO) and non-covalent interactions (NCI) 20

Calculated NMR and NICS 23

HOMA Analysis for Cyclopropane Derivatives 25

Scanning Tunneling microscopy (STM) 30

Single Crystal X-Ray Crystallographic (XRD) 31

Electron paramagnetic resonance (EPR) 37

Nuclear magnetic resonance spectroscopy (NMR) 38

Referances 40

General Information

Research Data Availability: Data from measurements and quantum chemical calculations can be found on Refubium DOI: http://dx.doi.org/10.17169/refubium-50747.

Reagents and Solvents: Unless otherwise specified, all reagents and solvents were used as supplied by commercial vendors. An MB-SPS-800 solvent purification system (Braun) was employed for the purification of dry solvents such as THF. Acetonitrile was dried by treatment with a sodium hydride dispersion for 10 minutes, distilled under argon, and subsequently stored in the dark over 3 Å molecular sieves.

Reaction Conditions: All reactions were conducted under an argon atmosphere unless stated otherwise. Glassware was subjected to evacuation, flame-drying, and purged with argon three times. Heating was carried out in an oil bath, with reported temperatures corresponding to the bath temperature. Cooling was achieved using either acetone/dry ice or water/ice mixtures, and the indicated values also refer to the bath temperature. Compound names were assigned according to ChemDraw nomenclature, which may deviate from IUPAC conventions. Structural depictions were prepared with ChemDraw (version 21.0.0.28).

Chromatographic Techniques: Flash column chromatography was performed using silica gel M60 (40–63 µm, Macherey-Nagel). Thin-layer chromatography (TLC) was carried out on ALUGRAM® Xtra SIL G/UV254 plates coated with silica gel (Macherey-Nagel). Spots of analytes were detected under UV irradiation at 254 or 365 nm, or by staining with a permanganate solution (2.00 g $KMnO_4$ and 10.0 g K_2CO_3 in 200 mL H_2O). Preparative TLC (PTLC) was conducted on silica gel plates, 2 mm in thickness (SIL G 200, Carl Roth).

Nuclear magnetic resonance (NMR) Spectroscopy: ^{1}H and ^{13}C NMR spectra were acquired on JEOL ECX 400 (400 MHz), Bruker Avance 500 (500 MHz), JEOL ECZ 600 (600 MHz), or Bruker Avance 700 (700 MHz) spectrometers. Spectral analysis was performed using MestReNova software (version 14.2.3-29241)[1]. Chemical shifts (δ) are given in ppm relative to the corresponding deuterated solvent and coupling constants (*J*) are reported in hertz (Hz). The deuterated solvents $CDCl_3$, DMSO-d_6, CD_3OD, and CD_3CN were used, with residual solvent signals used as internal references ($CDCl_3$: δ = 7.26 ppm in ^{1}H NMR, 77.16 ppm in ^{13}C NMR; DMSO-d_6: δ = 2.50 ppm in ^{1}H NMR, 39.52 ppm in ^{13}C NMR; CD_3OD: δ = 4.87 or 3.31 ppm in ^{1}H NMR, 49.00 ppm in ^{13}C NMR; CD_3CN: δ = 1.94 ppm in ^{1}H NMR, 118.26 or 1.32 ppm in ^{13}C NMR)[2]. Signal multiplicities are abbreviated as s (singlet), d (doublet), t (triplet), q (quartet), quint (quintet), sext (sextet) and br (broad).

Electron paramagnetic resonance (EPR) spectra: Spectra were recorded on a *Magnettech ESR5000* spectrometer (Bruker, Germany) operating at the X-band frequencies (~9.48 GHz). Measurements were performed either in solution (MeCN) or in the solid state, at room temperature (295 K) or at liquid nitrogen temperature (77 K). Spectral simulations were carried out using the *EasySpin* toolbox (version 6.0.10) implemented in MATLAB (R2025a).[3]

Mass Spectrometry: High-resolution mass spectra were recorded using either an ESI-FTICR-MS (Ionspec QFT-7, Varian Inc.) or an HR-EIMS instrument (Autospec Premier, Waters).

Cyclic voltammetry (CV): Measurements were performed under argon or nitrogen atmosphere in a dry and degassed solution of MeCN with tetra-*n*-butylammonium hexafluorophosphate (0.1 M) containing a concentration of $c = 10^{-3}$ mol/L analyte. For the measurements either a setup of a glassy carbon working electrode with a Ag/Ag^{+} reference electrode and platin counter electrode or for the different scan rates and the Differential pulse voltammogram (DPV) a Reference and working electrode with a platinum counter electrode was used with Fc/Fc^{+} as reference.

Infrared (IR) Spectroscopy: IR-spectra were measured on a PerkinElmer Spectrum Two FT-IR spectrometer equipped with a $LiTaO_3$ detector, also capable of measuring transition IR-spectra.

UV/Vis Spectroscopy: UV/Vis spectra were measured on a Perkin Elmer LAMBDA 365 UV/Vis Spectrophotometer with an Si diodes detector using cuvettes with 10 mm path length.

Fluorescence Spectroscopy: Fluorescence spectra were recorded on a Perkin Elmer FL 6500 fluorescence spectrophotometer equipped with an R928 PMT detector, using cuvettes with a 10 × 10 mm path length.

Data obtained from IR, UV/Vis, and fluorescence measurements were processed and analysed with Origin software (version 2022).

Fluorescence Quantum Yield: Fluorescence quantum yields (Φ_{fl}) were measured using a Hamamatsu Quantaurus-QY C11347-11 system equipped with an integrating sphere. All experiments were performed at room temperature (RT). For solution measurements, 3 mL of dye solution was placed in a 10 × 10 mm long-neck quartz cuvette (Hamamatsu). For solid-state measurements, the intact (unground) compound was placed in a Hamamatsu quartz bowl. Reported Φ_{fl} values represent the average of four independent measurements and are rounded accordingly. Yields below 1% are given as <1% due to accuracy limitations. For absolute Φ_{fl} values above 10%, the measurement uncertainty is approximately ±5%. Excitation wavelengths were chosen to correspond to the absorption maxima.[4]

X-ray Diffraction (XRD): Single-crystal XRD measurements were performed on single crystals picked at room temperature and mounted on a 0.15 mm MiTeGen micromount using perfluoroether oil. Crystal data were collected on a Bruker D8 Venture diffractometer equipped with a Photon II area detector using CuKα radiation or MoKα at 100(2) K. Multiscan or TWINABS absorption correction was used as implemented in APEX IV (SADABS-2016/2). The structures were solved using the SHELXT[5] structure solution program with intrinsic phasing and refined by full-matrix least-squares methods using SHELXL, as implemented in SHELXLE. Hydrogen atoms at carbon atoms were refined using a riding model with HFIX 137 (CH3), HFIX 43 (Csp2H) as implemented in SHELXL.

Scanning tunnelling microscopy: CN6CP molecules were sublimed under ultra-high vacuum onto a Cu(111) substrate held at room temperature, which had been cleaned by repeated sputtering-annealing cycles. The sample was then cooled down and transferred in vacuo into a scanning tunnelling microscope operating at a temperature of 1.3 K. The images were taken in constant-current mode with the parameters indicated in the figure captions.

Synthetics Procedures

Disodium 2,2',2''-(cyclopropane-1,2,3-triylidene)trimalononitrile

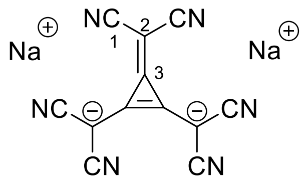

$C_{12}N_6Na_2$
M: 274.15 g/mol

NaH (60 % dispersion in mineral oil, 3.56 g, 89.0 mmol, 6.33 eq.) was added to dry THF (100 mL) and cooled to -30 °C. Malononitrile (2.83 g, 42.9 mmol, 3.05 eq.) was added in small portions, upon which vigorous gas evolution was observed. The suspension was stirred for 15 min. At the same temperature, TCCP (1.72 mL, 14.1 mmol, 1.0 eq.) in dry THF (10 mL) was added dropwise to the suspension. The reaction mixture was stirred for 30 min and then allowed to warm to 0 °C over 1 h. The reaction turned from yellow to beige. Brine (10 mL) was added, and the reaction mixture was filtered. The pale grey solid was recrystallised from water (150 mL) under continuous stirring to prevent the formation of a waxy, non-crystalline material. The precipitation was collected by filtration and washed with a small amount of ice-cold water. Drying yielded the product (2.63 g, 9.59 mmol, 68%) as pale grey, very thin, thread-like crystals.

^{1}H NMR (126 MHz, DMSO-d_6): no resonances attributable to impurities were observed.

^{13}C NMR (126 MHz, DMSO-d_6): δ[ppm] = 124.6 (C3), 121.1 (C1), 24.9 (C2) ppm.

IR (FT): $\tilde{\nu}$ [cm^{-1}] = 2205 (CN), 2173 (CN), 1428 (CP), 1147, 566.

Raman (λ = 532 nm, solid on SiO_2): $\tilde{\nu}$ [cm^{-1}] = 2309, 2262, 2013, 1965, 1514.

HRMS (ESI): m/z = (cal. $[C_{12}N_6Na]^-$ ($[M-Na]^-$): 251.0087; found 250.9594, (cal. $[C_{12}N_6]^-$ ($[M-2Na]^-$): 228.0189; found 227.9717, (cal. $[C_{12}N_6]^{2-}$ ($[M-2Na]^{2-}$): 114.0097; found 113.9759.

UV/Vis (MeCN): λ_{max} [nm] = ε [$L \cdot mol^{-1} \cdot cm^{-1}$] = 222 ($\varepsilon$ = 23200), 317 (ε = 21700).

T_{Smp}: mp > 300 °C.

EA: ($C_{12}N_6Na_2$) C: 52.57%, N: 30.66%, H: 0.0%; found C: 52.60%, N 30.42%, H: 0.210%.

Potassium 2,2',2''-(cyclopropane-1,2,3-triylidene)trimalononitrile anion radical

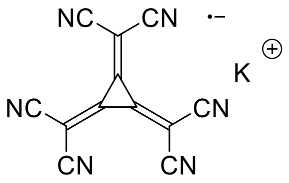

$C_{12}KN_6^{\bullet}$
M: 267.27 g/mol

$K_2S_2O_8$ (2.96 g, 10.9 mmol, 1.5 eq.) was dissolved in water (100 mL) and **$Na_2^+[(CN)_6CP]^{2-}$** (2.00 g, 7.30 mmol, 1.0 eq.) was added in small portions. Immediately, a colour change to blue/purple occurred. The reaction was stirred for 2 h at RT and bronze-coloured small crystals formed. The suspension was filtered through a glass frit (P4), was washed with water (2 x 3 mL) and dried under reduced pressure. **K^+[CN6CP]**$^{\cdot-}$ (1.84 g, 6.88 mmol, 94%) was obtained as purple-bronze, shimmering crystals.

EPR (X-Band *n* = 9.46 GHz, MeCN, 22 °C) = g_{iso} 2.00267, A(^{14}N) = 2.48 mT, ΔB_{pp} = 0.042; (P_{mw} = 10 mW, B = 335–340 mT, Mod = 0.05 mT @ 100 kHz, Sweep = 60 s).

^{1}H NMR (126 MHz, DMSO-d_6): δ[ppm] = no resonances attributable to impurities were observed.

^{13}C NMR (126 MHz, DMSO-d_6): δ[ppm] = no resonances attributable to impurities were observed.

IR (FT): $\tilde{\nu}$ [cm^{-1}] = 2928, 2214 (CN), 1814, 1484 (CP ν_{as}), 575.

IR (transmission, ZnSe): $\tilde{\nu}$ [cm^{-1}] = 2928, 2213 (CN), 1483 (CP ν_{as}), 1469, 898.

Raman (λ = 532 nm, solid on SiO_2): $\tilde{\nu}$ [cm^{-1}] 2218 (CN), 1848 (CP, ν_s), 1471 (CP, ν_{as}).

HRMS (ESI): m/z = (cal. $[C_{12}N_6]^-$ ($[M-K]^-$): 228.0189; found 227.9768, (cal. $[C_{24}N_{12}K]^-$ ($[2M-K]^-$): 495.0011; found 494.9353.

UV/VIS (MeCN): λ_{max} [nm], ε [L·mol^{-1}·cm^{-1}] = 213 (ε = 15500), 321 (ε = 19100), 598 (ε = 7600), 673 (ε = 11100).

T_{Smp}: decomposition > 280 °C.

EA: ($C_{12}N_6K$) C: 53.93%, N: 31.44%, H: 0.0%; found C: 52.27%, N 30.35%, H: 1.260%.

XRD: see **Figure S19**.

Tetrabutylammonium 2,2',2''-(cyclopropane-1,2,3-triylidene)trimalononitrile anion radical

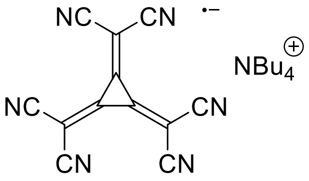

$C_{28}H_{36}N_7^{\bullet}$
M: 470.65 g/mol

To a suspension of **K^+[CN6CP]$^{\cdot-}$** (300 mg, 1.12 mmol, 1.0 eq.) in MeCN (25 mL), NBu_4Br (362 mg, 1.12 mmol, 1.0 eq.) was added. The suspension was stirred for 10 min. Subsequently, the precipitated salt was filtered off and washed with a small amount of MeCN. The solvent was removed under reduced pressure. The solid was suspended in water, stirred for 30 min, filtered off and washed extensively with water. The solid was dried under reduced pressure to obtain **NBu_4^+[CN6CP]$^{\cdot-}$** (513 mg, 1.09 mmol, 97 %) as blue-bronze, shimmering solid.

EPR (X-Band *n* = 9.46 GHz, MeCN, 22 °C) = g_{iso} 2.00265, A(^{14}N) = 2.48 mT, ΔB_{pp} = 0.042; (P_{mw} = 10 mW, B = 335–340 mT, Mod = 0.05 mT @ 100 kHz, Sweep = 60 s).

^{1}H NMR (126 MHz, DMSO-d_6): δ[ppm] = 3.16 (m, 8H), 1,57 (m, 8H), 1.31 (q, *J* = 7.1 Hz, 8H), 1.94 (t, *J* = 7.2 Hz, 12H).

^{13}C NMR (126 MHz, DMSO-d_6): δ[ppm] =57.5, 23.1, 19.2, 13.5.

IR (transmission, ZnSe): $\tilde{\nu}$ [cm^{-1}] = 2962, 2875, 2181, 1485, 1412, 882, 741.

Raman (λ = 532 nm): $\tilde{\nu}$ [cm^{-1}] 2218 (CN), 1848 (CP ν_s), 1471 (CP ν_{as}).

HRMS (ESI): m/z = (cal. $[C_{28}H_{36}N_7]^-$ ([M]$^-$): 470.3032; found 470.3047, (cal. $[C_{12}N_6]^-$ ([M-NBu$_4$]$^-$): 228.0190; found 228.0192.

UV/Vis (MeCN): λ_{max} [nm], ε [L·mol^{-1}·cm^{-1}] = 318 (ε = 21400) 595 (ε = 8300), 672 (ε = 12400).

T_{Smp}: decomposition > 270 °C.

2,2',2''-(cyclopropane-1,2,3-triylidene)trimalononitrile

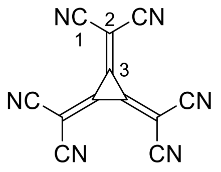

$C_{12}N_6$
M: 228.17 g/mol

In an inert atmosphere, the radical anion **K+[CN6CP]·−** (500 mg, 1.06 mmol, 1.0 eq.) was dissolved in dry MeCN (60 mL) and TFA (20 mL) at −40 °C. $NOSbF_6$ (283 mg, 1.06 mmol, 1.0 eq.) was dissolved in MeCN/TFA (3:1, 5 mL) and added dropwise to the solution. A dark yellow-orange suspension formed and was stirred for 1 h. Afterwards, the precipitate was filtered under argon, washed with TFA (3 x 2 mL) and dried under reduced pressure for 24 h. The molecule crystallizes with one equivalent of MeCN, making drying challenging. Small orange crystals (203 mg, 0.890 mmol, 84 %) were obtained.

Purification and single crystals was achieved by adding **CN6CP** to a mixture of MeCN/TFA 4:1 with a small amount of dissolved $NOSbF_6$ and heated to reflux. Afterwards, the solution was filtered off if not fully dissolved and some small seed crystals were added. At −25 °C crystals big enough for XRD formed over a period of one week. By adding a small amount of $NOSbF_6$ the crystals in solution are stable for at least several weeks. If dried, they tend to decompose very slowly to the corresponding radical anion even under argon at −30 °C. The compound reacted with every nucleophilic solvent tested (but slowly with MeCN or $MeNO_2$) but was insoluble in other solvents (e.g. DCM, H_2SO_4, TFA, hexane, ...).

^{1}H NMR (300 MHz, solid): traces from MeCN.

^{13}C NMR (300 MHz, solid): δ[ppm] = 135.5 (C-3), 118.2-113.6 (C1), 85.1 (C2).

IR (FT): $\tilde{\nu}$ [cm^{-1}] = 2219, 1564, 1220, 1060, 607, 575.

IR (transmission, ZnSe): $\tilde{\nu}$ [cm^{-1}] = 2925, 2852, 2220, 1564, 1221, 1060, 620.

MS (ESI): m/z = (cal. $[C_{12}N_6]^-$ ($[M-K]^-$): 228.02; found 228.00, (cal. $[C_{12}N_6MeO]^-$ ($[M+MeO]^-$): 259.13; found 259.13.

Raman (λ = 532 nm, solid on ZnSe): $\tilde{\nu}$ [cm^{-1}] = 1098, 1472, 1736, 1768 (CP), 1808, 2228 (CN).

UV/Vis ($MeNO_2$, saturated): λ_{max} [nm] = 420, 447, 484.

Emission ($MeNO_2$, saturated): λ_{max} [nm] = 568.

T_{Smp}: slow decomposition at room temperature and fast decomposition above 130 °C.

XRD: see **Figure S21**.

Spectroscopic Measurements

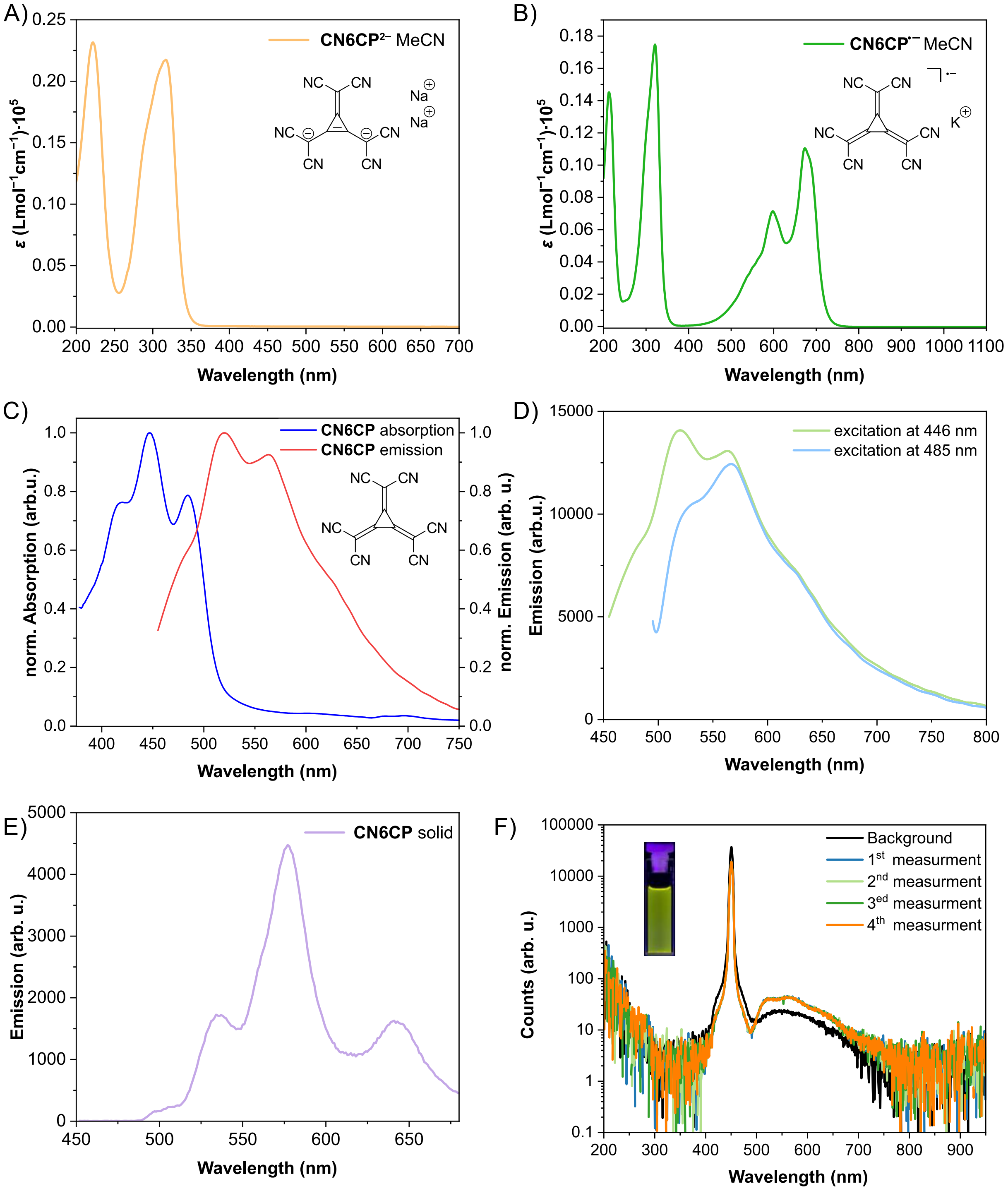

Figure S1: A) UV/Vis spectra of **CN6CP^{2-}** (orange). **B**) UV/Vis spectra of the radical anion **CN6CP$^{\cdot-}$**. **C**) Normalised UV/Vis spectra (blue) and emission spectra (red) (ex = 446 nm) of a saturated solution of **CN6CP** in MeCN/TFA (3:1) excited at 446 nm. **D**) Emission spectra of **CN6CP** in MeCN/TFA (3:1) excited at 446 nm (pale green) and at 485 nm (pale blue). **E**) Emission spectra of solid **CN6CP**. **F**) Fluorescence quantum yield (ϕ_{fl}) measurement of **CN6CP** in a saturated solution of MeCN/TFA (3:1). A ϕ_{fl} of 5% was determined. The compound was not stable enough for a lifetime measurement in this solvent.

Cyclic voltammetry

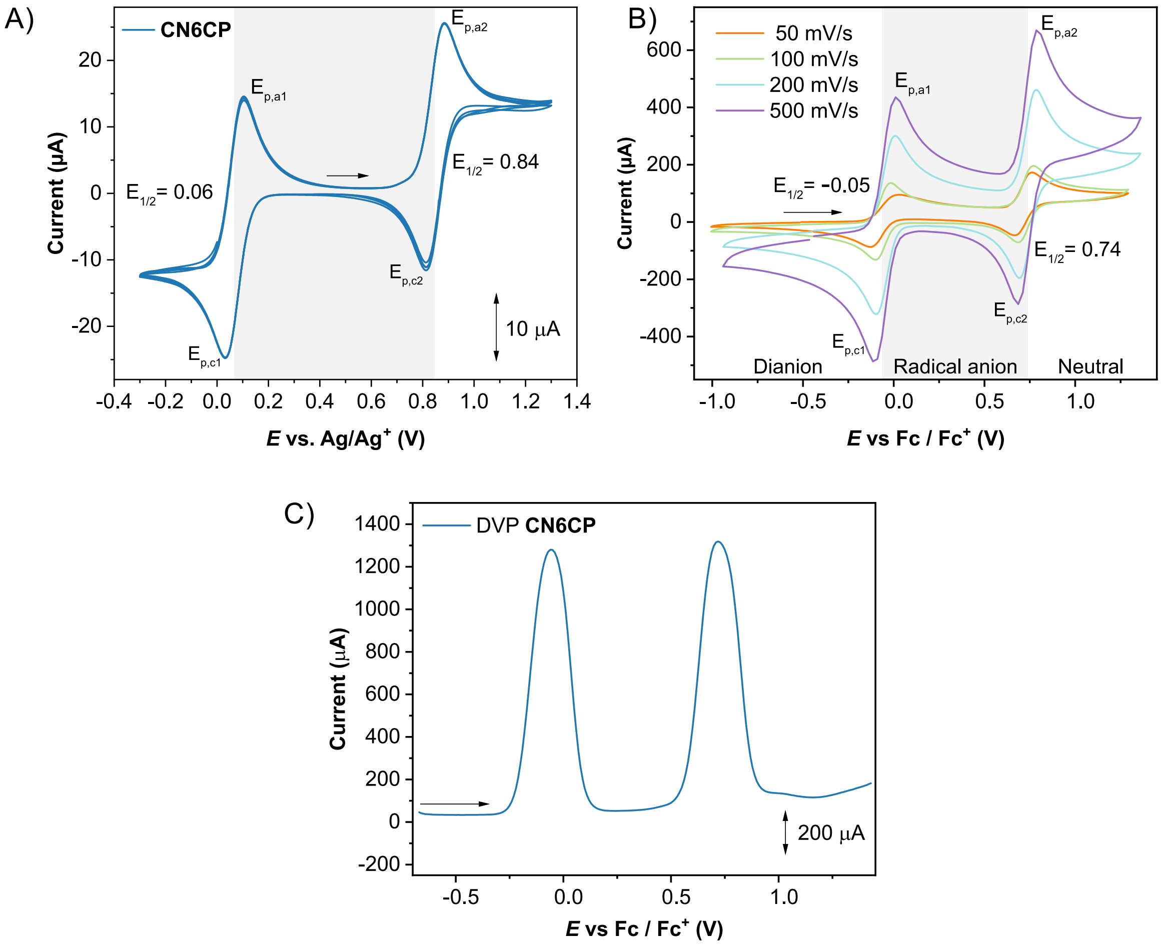

Figure S2: A) Cyclic voltammogram of a 1 mM solution of **CN6CP** in MeCN with 0.1 M NBu_4PF_6, recorded at a scan rate of 100 mV/s. The second to fifth cycles are shown, demonstrating reversible behaviour. **B)** Cyclic voltammograms of a 0.1 mM solution of **CN6CP** with 0.1 M NBu_4PF_6 in MeCN at different scan rates 50 mV/s (orange), 100 mV/s (green), 200 mV/s (blue) and 500 mV/s (purple). **C)** Differential pulse voltammogram of a 0.1 mM solution of **CN6CP** with 0.1 M NBu_4PF_6 in MeCN recorded with an effective scan rate of 20 mV/s.

For **Figure S2 A**: The peak-to-peak potential separation ΔE_p is approximately 74 mV ($E_{p,a}$(first ox.)−$E_{p,c}$(first ox.)) and approximately 79 mV for $E_{p,a}$(second ox.)−$E_{p,c}$(second ox.), close to the ideal 59 mV for a scan rate of 100 mV/s.[6] The ratio (i_{pa}/i_{pc}) is close to one (1.04 and 1.06) and was determined with the Cyclic Voltammetry (Pro) add-on for Origin 2026, indicating a reversible redox-reaction.

Table S1: Electrochemical properties from the cyclic voltammetry measurement of a 0.1 mM solution of **CN6CP** with 0.1 M NBu_4PF_6 in MeCN at a scan rate of 100 mV/s referenced against Fc/Fc$^+$.

	E_{pa} (V)	E_{pc} (V)	E_{pa}-E_{pc} (mV)	I_{pc}/I_{pa}	$E_{1/2}$(V)
1. ox.	-0.084	-0.010	74	1.04	-0.05
2. ox.	0.776	0.699	77	1.06	0.74

The calibration of the electrochemical potentials was performed using the ferrocene/ferrocenium redox couple as internal reference. The formal potential of Fc/Fc^+ is commonly taken as $E_0' \approx E_{1/2} = 0.40$ V vs the saturated calomel electrode (SCE). Considering the standard conversion between SCE and the normal hydrogen electrode (NHE) of +0.25 V, this corresponds to a potential of 0.65 V versus NHE.

To convert electrochemical potentials into absolute energy levels, the Fermi level was referenced according to the scale proposed by Trasatti, where 0.0 V versus NHE is equivalent to −4.44 eV on the absolute energy scale. On this basis, the Fc/Fc^+ redox level is located at −5.09 eV. This value was used as the reference point for estimating frontier orbital energies from cyclic voltammetry data.

Accordingly, for molecular dopants exhibiting electrochemically reversible reduction processes, the LUMO energy levels were calculated from the half-wave reduction potentials according to:

$$E_{\mathrm{LUMO}} = -(5.09 + E_{1/2,\,\mathrm{red}} \text{ vs. Fc/Fc}^{+})\ \mathrm{eV} \qquad \text{Eq. 1}$$

	CV		Calculated
	$E_{1/2,\,\mathrm{red}}$	E_{LUMO}	ΔE (KS-Gap)
**CN6CP**	0.74 V	-5.85 eV	6.61 eV
**CN6CP**$^{\cdot-}$	-0.05 V	-5.05 eV	9.06 eV

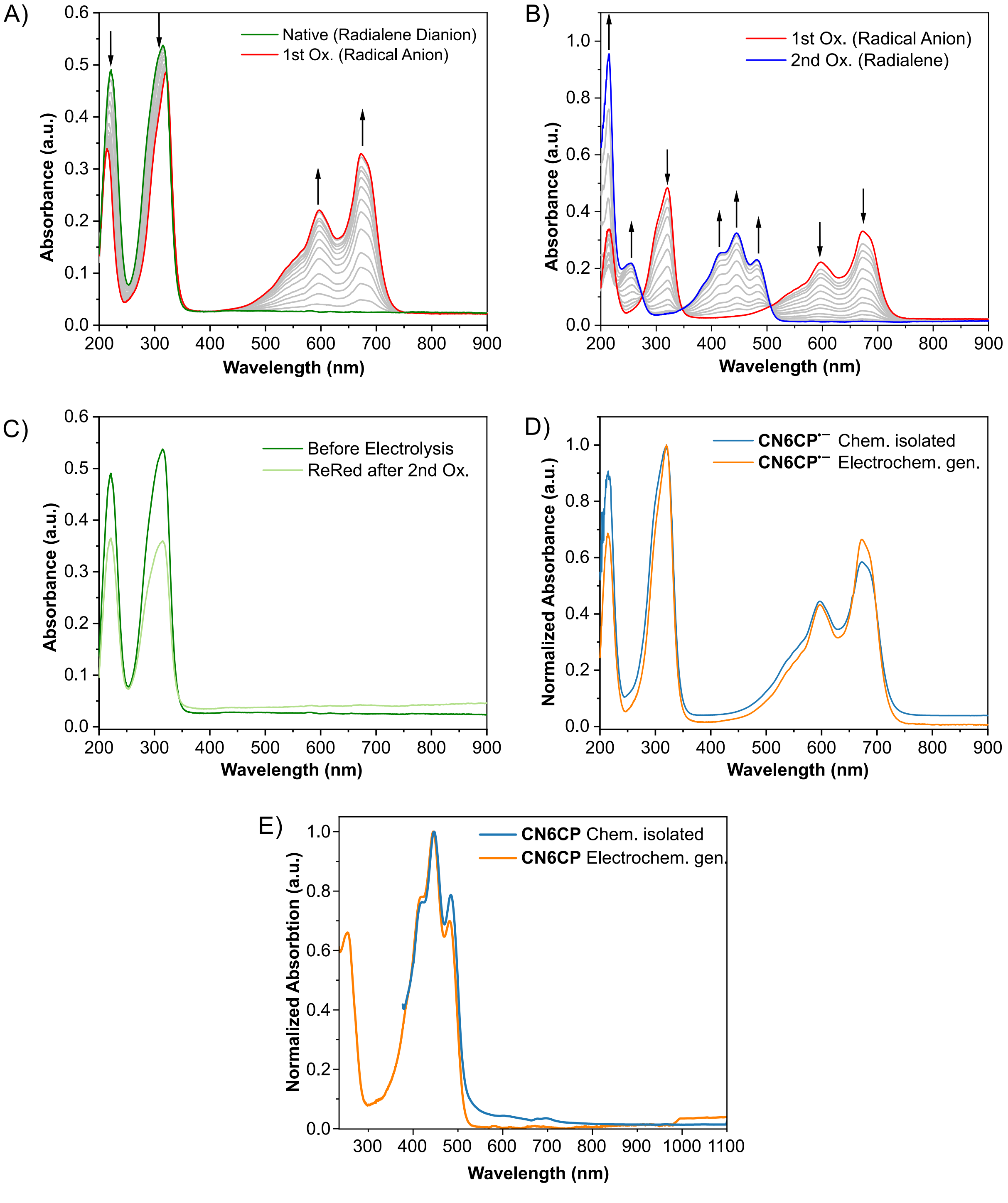

Figure S3: A) Changes in the UV/Vis/NIR spectra of **CN6CP** in MeCN/0.1 M Bu_4NPF_6 during the 1st oxidation with an Au working electrode. **B)** Changes in the UV/Vis/NIR spectra of **CN6CP** in MeCN/0.1 M Bu_4NPF_6 during the 2nd oxidation with an Au working electrode. **C)** The UV/Vis/NIR spectra of native and re-reduced form of **CN6CP** after 2nd oxidation in MeCN/0.1 M Bu_4NPF_6. **D)** UV/Vis spectra of the chemically isolated (blue) and electrochemically generated (orange) radical anion **CN6CP**$^{\bullet-}$. A close match is observed. **E**) UV/Vis/NIR spectra of the chemically isolated (blue) and electrochemically generated (orange) neutral **CN6CP**. The chemically generated **CN6CP** was measured in a MeCN/TFA (3:1) mixture, which limits the optical window below approximately 380 nm.

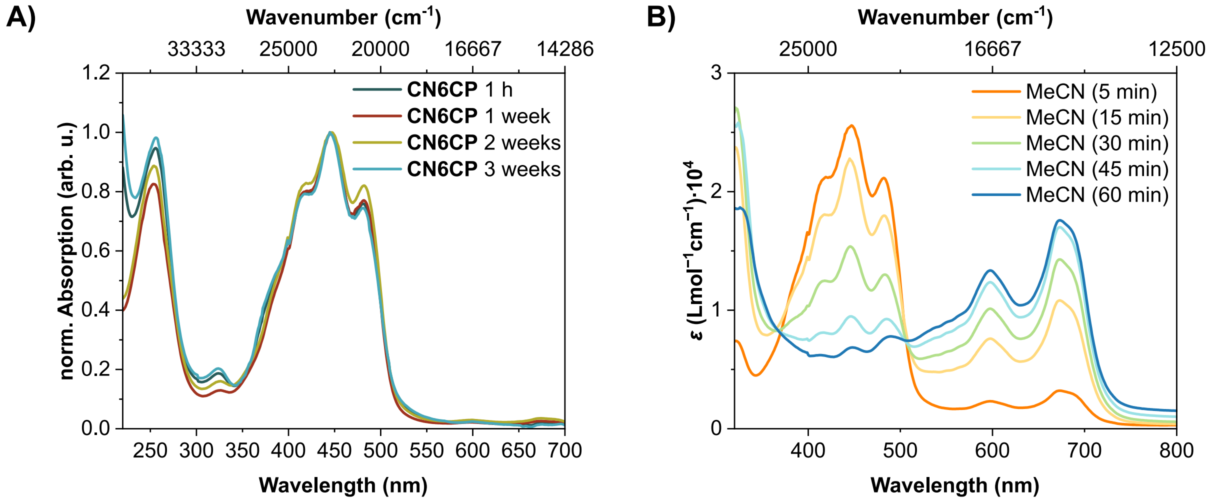

Figure S4: **A)** UV/Vis spectra of freshly dissolved crystals of **CN6CP** in MeCN/TFA (4:1). Following synthesis, the crystals were stored in a MeCN/TFA (4:1) solution containing a small amount of $NOSbF_6$. Overall, no clear evidence of crystal degradation was observed over a period of 3 weeks. **B)** UV/Vis spectra of a solution of **CN6CP** in MeCN recorded over 1 h, indicating that **CN6CP** mainly decomposes to the radical anion.

Thermogravimetric analysis (TGA)

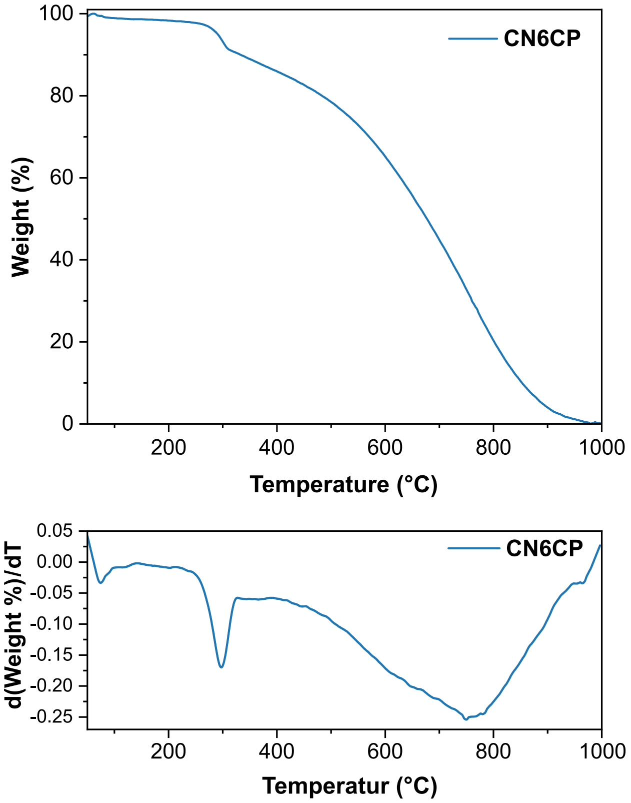

Figure S5: Top: integral and **bottom:** differential thermogravimetric analysis (TGA) of **CN6CP** under inert N_2 gas flow. The mass loss below 270 °C is attributed to the evaporation of solvent encapsulated within the crystals. After an initial mass loss of approximately 9%, a gradual and continuous decomposition process is observed, resulting in complete mass loss by 980 °C.

Computational Details

Quantum-chemical calculations were performed to analyse charge and spin distributions and to elucidate the electronic structure. Geometry optimisations were carried out with ORCA (version 6.1.0) [7, 8] at the ωB97X-D3/def2-TZVPD level of theory.[9, 10] Acetonitrile ($\varepsilon = 37.5$ at 20 °C) or DMSO ($\varepsilon = 46.8$ at 20 °C)[11] was included via the conductor-like polarisable continuum model (CPCM) implicit solvation model.[12] ORCA's default convergence criteria (e.g, an energy change threshold of 5 µHartree) were applied throughout.

The ωB97X-D3 functional was selected because of its robust performance for dianions and radical species; its range-separated hybrid design effectively reduces self-interaction errors and therefore provides a more reliable description of charge distribution and spin localisation.[13]

To further probe the aromatic character of **CN6CP**, nuclear-independent chemical shift (NICS) values were computed. These calculations were performed using Gaussian16[14] at the ωB97X/def2-TZVPD[9, 10] level of theory, employing DMSO as the solvent by using the CPCM implicit solvation model.

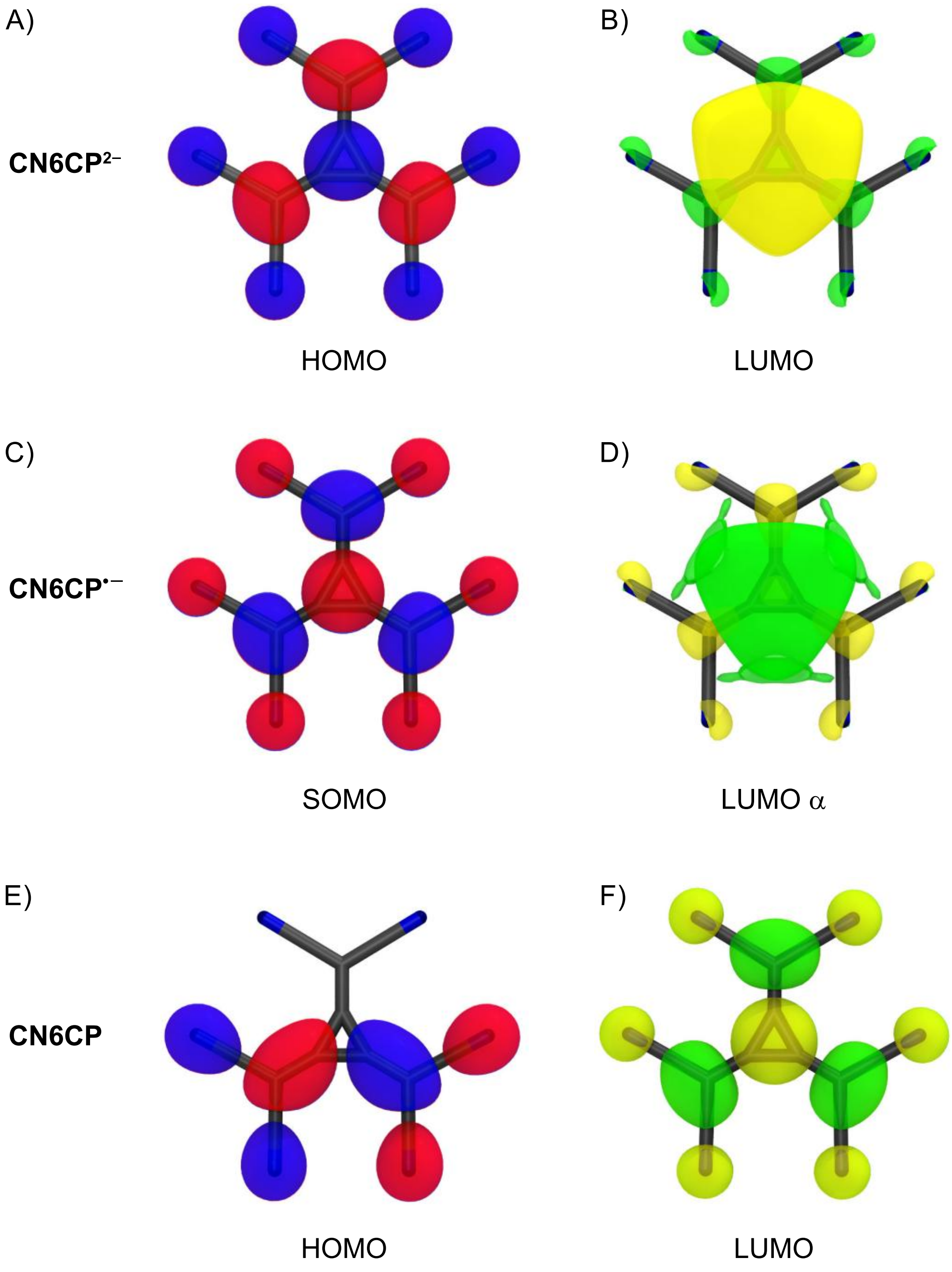

Figure S6: Highest occupied molecular orbitals (HOMOs) and lowest unoccupied molecular orbitals (LUMOs) of the dianion (**A** and **B**), single occupied molecular orbital and LUMO α of the radical anion (**C** and **D**), as well as HOMO and LUMO of the neutral **CN6CP** (**E** and **F**). The orbitals were calculated at the ωB97D3 level using the def2-TZVPD basis set, employing the RIJCOSX approximation with the def2/J auxiliary basis and a conductor-like polarisable continuum model (CPCM) for solvation in acetonitrile. All orbitals were shown as isosurfaces enclosing 80% of the integrated electron probability density ($|\Psi|^2$).

A) B)

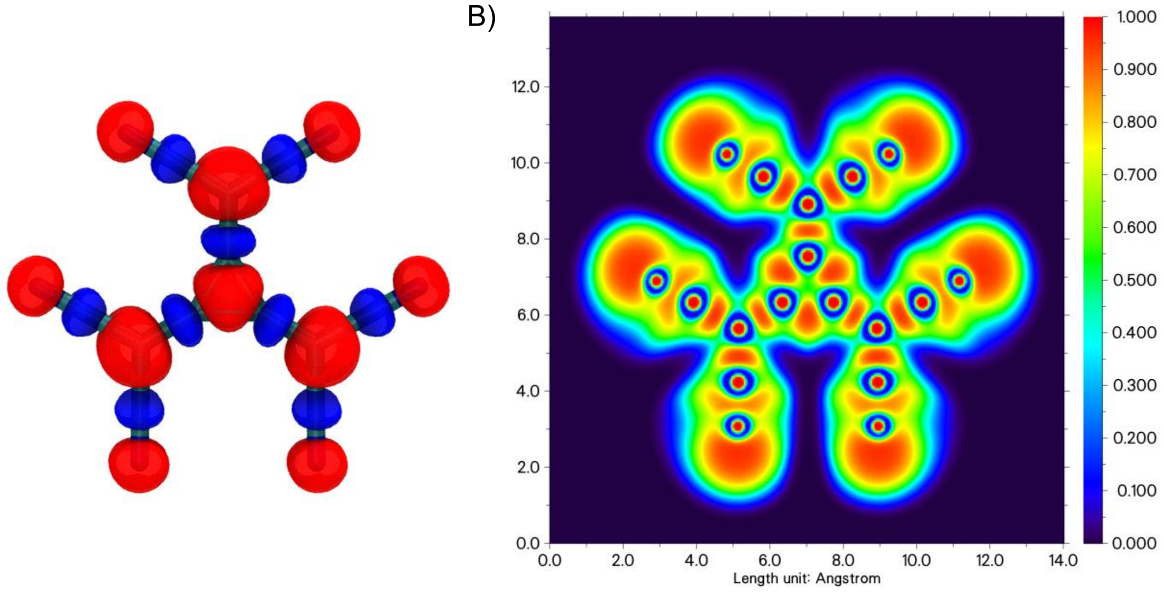

Figure S7: A) Spin density of the radical anion **CN6CP**$^{\cdot-}$ depicted using VMD calculated at the ωB97D3 level with the def2-TZVPD basis set, employing the RIJCOSX approximation (def2/J auxiliary basis) and a CPCM solvation model for acetonitrile using ORCA 6.0. **B**) Electron localisation function (ELF) of the radical anion (**CN6CP**$^{\cdot-}$) depicted in the XY-plane through the molecule. The values range from 0.0 (dark blue) to 1.0 (red). ELF were determined and graphically represented using Multiwfn 3.8 with a 200 x 200 grid.[15, 16] The structures have been optimised at ωB97D3 level with def2-TZVPD as basis set using ORCA 6.1.The ELF is very similar across all three oxidation states of **CN6CP**, as it is predominantly influenced by core electrons. –

Electron distribution

A) B) C)

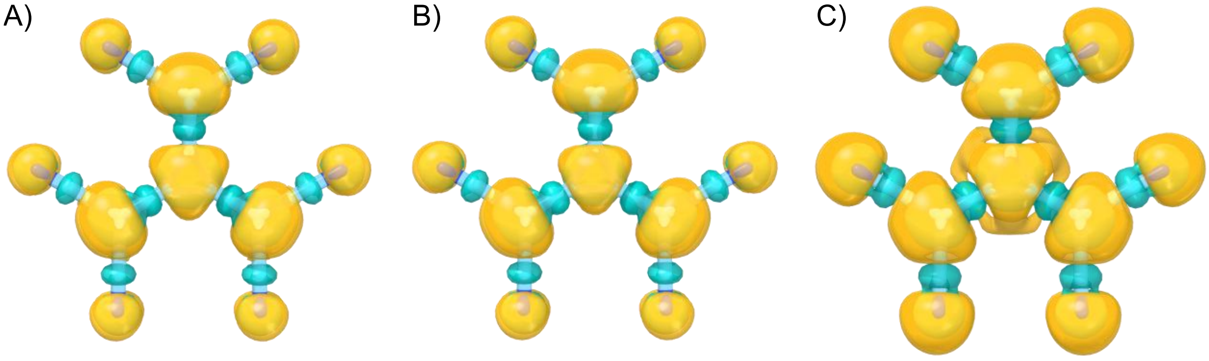

Δρ = ρ(Radical) – ρ(Neutral) Δρ = ρ(Dianion) – ρ(Radical) Δρ = ρ(Dianion) – ρ(Neutral)

Figure S8: Single-point DFT calculations were carried out using the ωB97X-D3 functional with the def2-TZVPD basis set, using the RIJCOSX approximation with the def2/J auxiliary basis and a CPCM solvation model for acetonitrile. Electron density difference maps ($\Delta\rho = \rho(X) - \rho(Y)$) were generated by subtracting the respective cube files in Multiwfn 3.8[15, 16] and plotted as isosurfaces at ±0.001 e bohr^{-3} in UCSF Chimera[17], using gold for regions of electron gain and turquoise for regions of electron loss. **A**) depicts the Electron density difference $\Delta\rho$ from the radical-anion (**CN6CP**$^{\cdot-}$) to neutral **CN6CP**, **B**) the $\Delta\rho$ from the Dianion (**CN6CP**$^{2-}$) and the radical anion (**CN6CP**$^{\cdot-}$) and **C**) the $\Delta\rho$ from the dianion (**CN6CP**$^{2-}$) to the neutral (**CN6CP**). Overall, the additional electron density (gold) is primarily located in π orbitals on the nitrile nitrogen atoms, the central carbon atom of the dicyanomethylene groups, as well as on the central cyclopropane ring. Conversely, electron density is lost within the molecular plane (turquoise).

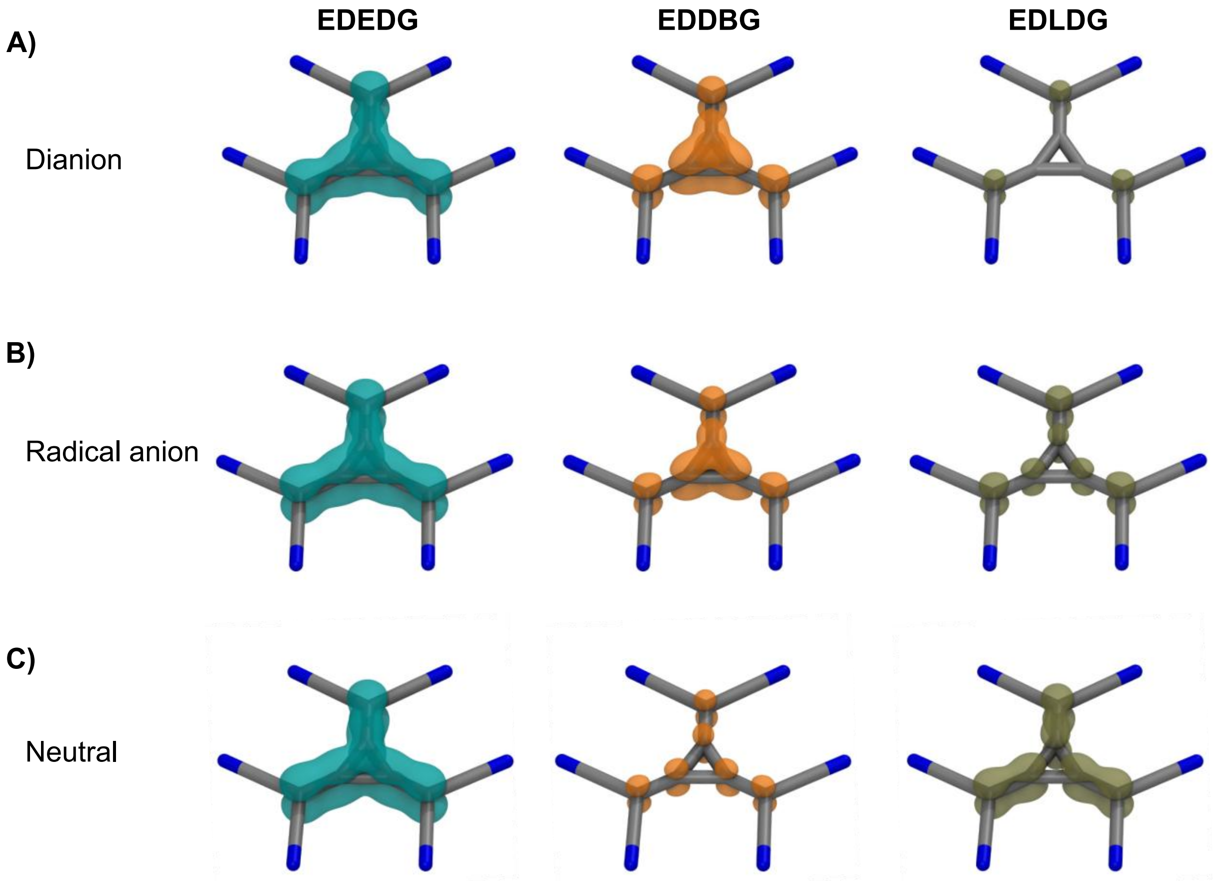

Figure S9: Depiction of the π-electron density using the electron density of electron delocalisation graph (EDEDG, turquoise), the electron density of delocalised bonds graph (EDDBG, orange) and the electron density of localised bonds graph (EDLDG, green) for all three oxidation states of **CN6CP**. Electron density distributions of delocalised and localised π-electrons (EDEDG, EDDBG, and EDLDG) were calculated at the ωB97X-D3 functional with the def2-TZVPD basis set and were analysed using Multiwfn,[15, 16] and the resulting cube files were visualised using VMD 2.0. In contrast to literature-reported systems, **CN6CP** exhibits both localised and delocalised π-electron density on the central cyclopropane core as well as on the central carbon atoms of the dicyanomethylene substituents, while no π-electron density is observed on the nitrile groups. As the localised and delocalised π-electron densities are spatially distributed over the same atomic framework, a strict spatial separation between these contributions cannot be visualised. Such an overlap of localized and delocalized π-electron density contributions has been described previously within the EDDB/EDLD framework and reflects partial, rather than fully separated π-electron delocalisation, especially in non-classical and push-pull π systems.[15, 18]

Charge distribution

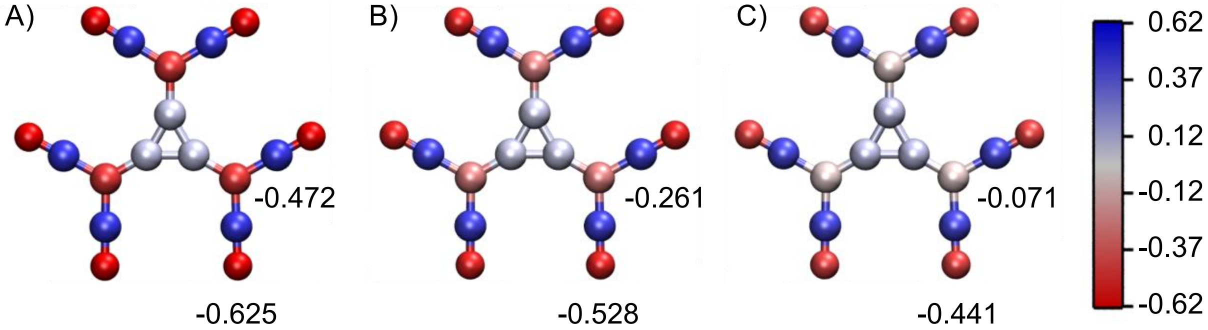

CN6CP^{2-} **CN6CP$^{\bullet-}$** **CN6CP**

Figure S10: Visual representation of the minimal basis iterative stockholder (MBIS) values, red indicates negative charges, white neutral and blue positive charges, **A**) The dianion, **B**) radical anion and **C**) neutral **CN6CP**. The values were calculated using ORCA 6.1 at the ωB97D3 level with the def2-TZVPD basis set, using the RIJCOSX approximation with the def2/J auxiliary basis and a CPCM solvation model for acetonitrile. The negative charge is predominantly localised on the nitrogen atoms as well as on the central dicyanomethylene carbon atoms.

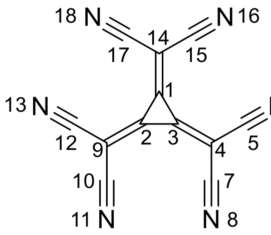

Figure S11: Assignment of atoms for charge and NMR analyses. The numbering is identical across all oxidation states.

Table S2: Charge analysis of the dianion (**CN6CP^{2-}**). Single-point calculations were performed at the ωB97X-D3/def2-TZVPD level, employing the RIJCOSX approximation with the def2/J auxiliary basis and a CPCM solvation model for acetonitrile.

AtomIndex	Element	Mulliken Charge	Loewdin Charge	MBIS Charge	ESP Charge	Hirschfeld
1	C	0.25182	-0.04312	0.09967	0.25975	0.01459
2	C	0.28313	-0.04212	0.09904	0.26543	0.0148
3	C	0.26237	-0.04262	0.09896	0.25726	0.01463
4	C	0.91867	-0.24915	-0.47241	-0.84579	-0.08462
5	C	-0.33745	-0.26376	0.4774	0.61781	0.04784
6	N	-0.58417	0.07684	-0.62376	-0.65852	-0.34568
7	C	-0.3393	-0.26366	0.47865	0.62359	0.04799
8	N	-0.58821	0.07585	-0.62554	-0.6633	-0.3467
9	C	0.90612	-0.24943	-0.47244	-0.85333	-0.08471
10	C	-0.33402	-0.26367	0.47835	0.62666	0.04793
11	N	-0.58832	0.07591	-0.62532	-0.66326	-0.34662
12	C	-0.33618	-0.26389	0.47766	0.62015	0.04777
13	N	-0.58669	0.07643	-0.62457	-0.66045	-0.34621
14	C	0.9189	-0.24906	-0.47255	-0.84523	-0.08458
15	C	-0.33449	-0.2639	0.47812	0.6219	0.04799
16	N	-0.58713	0.07691	-0.62432	-0.66166	-0.34591
17	C	-0.33664	-0.26376	0.47773	0.6195	0.04777
18	N	-0.5884	0.07618	-0.6247	-0.6605	-0.34633

Table S3: Charge analysis from the radical-anion (**CN6CP**$^{\bullet-}$). Charge and spin density are given. Single-point calculations were performed at the ωB97X-D3/def2-TZVPD level, employing the RIJCOSX approximation with the def2/J auxiliary basis and a CPCM solvation model for acetonitrile.

	E	Mulliken Charge	Mulliken Spin	Loewdin Charge	Loewdin Spin	MBIS Charge	MBIS Spin	ESP Charge	Hirsch-feld	Hirsch-Spin
1	C	0.070613	-0.02355	-0.00924	0.0147	0.091375	0.005576	0.190543	0.04328	0.00617
2	C	0.080428	-0.02342	-0.00916	0.01472	0.091038	0.00561	0.20205	0.04326	0.0062
3	C	0.06916	-0.02324	-0.00885	0.01467	0.091252	0.005523	0.196237	0.04337	0.00612
4	C	1.447178	0.106984	-0.17694	0.14245	-0.26071	0.177624	-0.48793	0.00726	0.17024
5	C	-0.40885	0.039172	-0.19751	0.01532	0.44606	-0.00906	0.536471	0.08641	-0.00442
6	N	-0.52339	0.08524	0.12388	0.07294	-0.527969	0.084289	-0.55691	-0.27827	0.08305
7	C	-0.40696	0.040513	-0.19725	0.01532	0.446997	-0.00903	0.535712	0.08675	-0.00439
8	N	-0.52028	0.085142	0.12373	0.0728	-0.52865	0.084133	-0.55684	-0.27854	0.08289
9	C	1.454905	0.105947	-0.17698	0.14224	-0.260724	0.177363	-0.49381	0.0071	0.16999
10	C	-0.40549	0.040302	-0.19743	0.01532	0.446623	-0.00902	0.540453	0.08655	-0.00438
11	N	-0.5223	0.085085	0.12373	0.07279	-0.52846	0.084117	-0.55819	-0.2785	0.08287
12	C	-0.41185	0.040143	-0.19771	0.01531	0.445466	-0.00901	0.534248	0.08618	-0.00438
13	N	-0.51899	0.085066	0.12403	0.07276	-0.527601	0.084089	-0.55524	-0.27815	0.08285
14	C	1.441474	0.106256	-0.17706	0.1423	-0.260968	0.177447	-0.48153	0.00707	0.17007
15	C	-0.39755	0.039502	-0.19728	0.0153	0.446805	-0.00907	0.534936	0.08664	-0.00442
16	N	-0.52326	0.085185	0.12383	0.07288	-0.528397	0.084229	-0.55665	-0.2784	0.08299
17	C	-0.40439	0.040472	-0.19778	0.01533	0.445405	-0.00902	0.530868	0.08612	-0.00439
18	N	-0.52045	0.085194	0.12401	0.07286	-0.527589	0.084199	-0.55441	-0.27817	0.08296

Table S4: Charge analysis from the neutral (**CN6CP**). Single-point calculations were performed at the ωB97X-D3/def2-TZVPD level, employing the RIJCOSX approximation with the def2/J auxiliary basis and a CPCM solvation model for acetonitrile.

AtomIndex	Element	Mulliken Charge	Loewdin Charge	MBIS Charge	ESPCharge	Hirschfeld
1	C	-0.1958	0.04543	0.1104	0.16878	0.08929
2	C	-0.17212	0.04632	0.11079	0.17187	0.08971
3	C	-0.19847	0.0459	0.11073	0.17367	0.08938
4	C	2.11156	-0.10302	-0.07127	-0.18849	0.09934
5	C	-0.51648	-0.14071	0.42057	0.47308	0.12143
6	N	-0.44896	0.16907	-0.44076	-0.46567	-0.21604
7	C	-0.51886	-0.13995	0.42268	0.47534	0.12219
8	N	-0.44857	0.16864	-0.44209	-0.46697	-0.21649
9	C	2.12673	-0.10323	-0.07039	-0.18709	0.09959
10	C	-0.50695	-0.14012	0.42211	0.47531	0.12209
11	N	-0.45131	0.16895	-0.44176	-0.46623	-0.21635
12	C	-0.52193	-0.14011	0.42087	0.47203	0.12166
13	N	-0.45008	0.16889	-0.44101	-0.46525	-0.2161
14	C	2.11283	-0.10343	-0.07113	-0.18519	0.09918
15	C	-0.49764	-0.1404	0.42155	0.47362	0.12182
16	N	-0.45008	0.1692	-0.44126	-0.46589	-0.21613
17	C	-0.52316	-0.14026	0.42109	0.4722	0.12158
18	N	-0.45071	0.16883	-0.44116	-0.46509	-0.21618

Bond Order calculations

To gain a deeper understanding of the **CN6CP** moiety we performed Bond order calculations on the ωB97XD/def2TZVP level including solvent effects with the polarizable continuum model (PCM) for acetonitrile. Overall neutral **CN6CP** can be well depicted as a [3]radialene framework with dicyanomethylene on the exocyclic bonds. However, the bond orders are with 1.56 slightly lower than expected for an [3]radialene (~1.75).

Container the dianion has a slightly higher bond order in the cyclopropane core and lower bond order on the exocyclic double bonds and can therefore be compared slightly more to a cyclopropenium core with three negatively charged dicyanomethylene substituents. This also ties in well with the stronger aromatic character observed in the dianion.

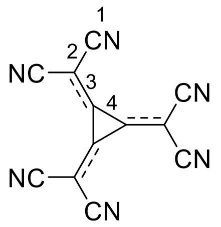

Table S5: Bond Order calculations obtained of **CN6CP** at the ωB97XD/def2TZVP level including solvent effects with the polarizable continuum model (PCM) for acetonitrile. The assignment of the bonds is depicted above this table.

Bonds	Dianion[19]	Radical anion	Neutral
1	2.775	2.826	2.867
2	1.142	1.100	1.070
3	1.200	1.351	**1.563**
4	**1.235**	1.160	1.083

Non-bonding orbitals (NBO) and non-covalent interactions (NCI)

To gain further insight into the crystal interacting forces non-bonding orbitals (NBOs) and non-covalent interactions (NCIs) were calculated. Second-order NBO calculation of a tetrameric unite cut from the XRD-structure indicate that is no single strong intermolecular NBO interaction, but rather a network of many small contributions. This suggests weak, dispersed connections, rather than a dominant, specific bond. Overall weak nitrile to nitrile contacts consistent with periplanar and shared antiparallel dipolar C≡N···C≡N interactions. Additionally weak hydrogen bonds between the co crystallised MeCN and **CN6CP** are observed.

Table S6: Summary of the most relevant noncovalent NBO interactions obtained from a tetramer fragment extracted from the X-ray diffraction structure and calculated at the ωB97XD/def2TZVP level including solvent effects with the polarizable continuum model (PCM) for acetonitrile. Overall, only weak van der Waals forces and weak hydrogen bonds from the co-crystalised MeCN towards **CN6CP** are observed.

Interface type	Donor NBO	Acceptor NBO	motive	$E^{(2)}$ (range) kcal/mol
CN6CP → CN6CP (antiparallel)	LP(N)	π*(C≡N)	n→ π*	~0.17–0.41
CN6CP → CN6CP (perpendicular)	LP(N)	π*(C≡N)	n→ π*	0.27
CN6CP → CN6CP	π(C≡N)	π*(C≡N)	π→π*	up to 0.18
CN6CP → MeCN	LP(N)	π*(C≡N)	n→ π*	~0.26–0.34
CN6CP → MeCN	σ(C–H)	π*(C≡N)	σ→π*	~0.21–0.23
CN6CP → MeCN	LP(N)	σ*(C–H)	n→σ*	~0.15–0.16

To gain a deeper understanding and to visualise the interactions we performed Non-covalent interaction (NCI) calculations. NCI analysis is a real-space method based on the electron density, $\rho(r)$, and the reduced density gradient, $s(r)$, which enables the visualization of weak intra- and intermolecular interactions. NCI regions are identified as low-gradient areas in the low-density regime and are commonly visualized as three-dimensional isosurfaces of $s(r)$. To distinguish between attractive and repulsive interactions, the isosurfaces are coloured according to $\mathrm{sign}(\lambda_2)\rho(r)$, where λ_2 is the second eigenvalue of the Hessian of the electron density. Negative values of $\mathrm{sign}(\lambda_2)\rho(r)$ indicate attractive interactions, values close to zero correspond to weak dispersive or van der Waals contacts, and positive values indicate steric repulsion. Accordingly, the NCI plots shown here provide a qualitative real-space representation of the location and nature of weak interactions rather than a direct measure of interaction energies.[20, 21]

In our case only weak interactions most likely manly Van der Waals forces or CN to CN dipolar interactions are observed between the **CN6CP** molecules and weak hydrogen bonds or Van der Waals forces are observed between the co-crystalised MeCN and **CN6CP**. The interactions are visualised in **Figure S12**.

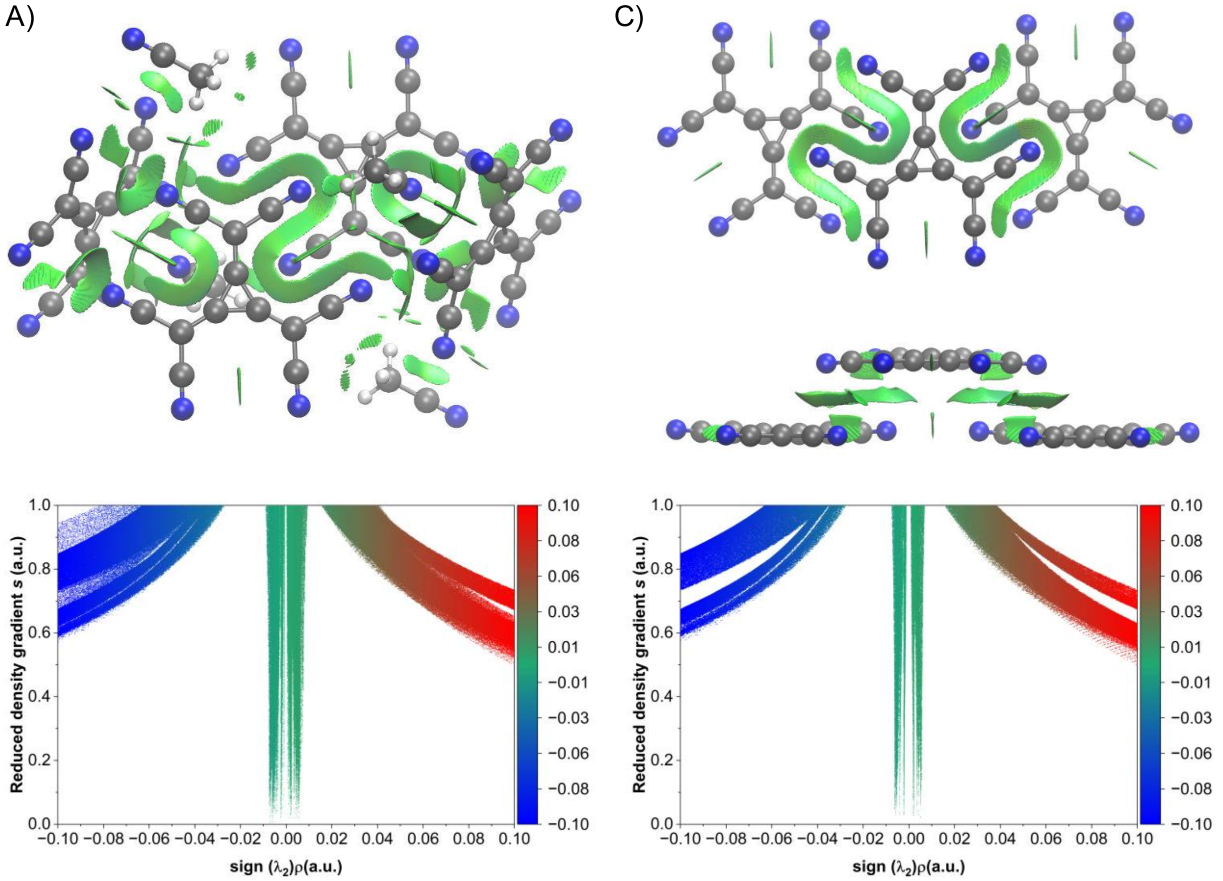

Figure S12: A) Tetramer of **CN6CP**·MeCN and **B)** trimer of **CN6CP**. Top: three-dimensional NCI isosurface, visualising weak interaction regions in real space, coloured according to sign(λ_2)ρ to distinguish attractive (blue), weak dispersive/van der Waals (green), and repulsive (red) interactions. Bottom: corresponding RDG scatter plot (*s* vs sign(λ_2)ρ), in which each point represents a grid point in space; negative values indicate attractive interactions, values near zero weak van der Waals contacts, and positive values steric repulsion.

Calculated NMR and NICS

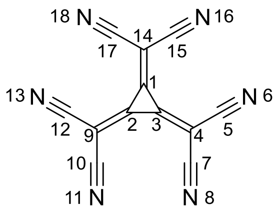

Table S7: Calculated NMR spectra for **CN6CP**$^{2-}$ and the neutral **CN6CP**. The chemical shifts were calculated at the ωB97D3 level with the def2-TZVPD basis set. The ^{13}C-TMS shielding was set to 184.025 ppm. A close agreement with the experimental spectra is observed.

^{13}C NMR	Dianion **CN6CP**$^{2-}$	Neutral **CN6CP**
C1	127.749	132.5269
C2	127.3015	132.4523
C3	127.5318	132.5229
C4	18.7909	86.1706
C5	125.2874	111.7493
C7	125.4822	111.7962
C9	18.606	86.1333
C10	125.6872	112.0732
C12	125.4767	111.9058
C14	18.8691	86.4882
C15	125.4757	111.9007
C17	125.4552	111.8825

1.3 NICS

NMR- and NICS-values were calculated at the ωB97D3 level with the def2-TZVPD basis set. The NMR and NICS values were calculated using the conductor-like polarisable continuum model (CPCM) for DMSO (ε = 46.8 at 20 °C) as the solvation model. For the generation of the ghost atoms the software package pyarome was used.[22] For the generation of the sigma only plots protons were placed in a 90 ° angle to the respective atoms plane with C-H distances of 1.09 Å and N-H distances of 1.01 Å.

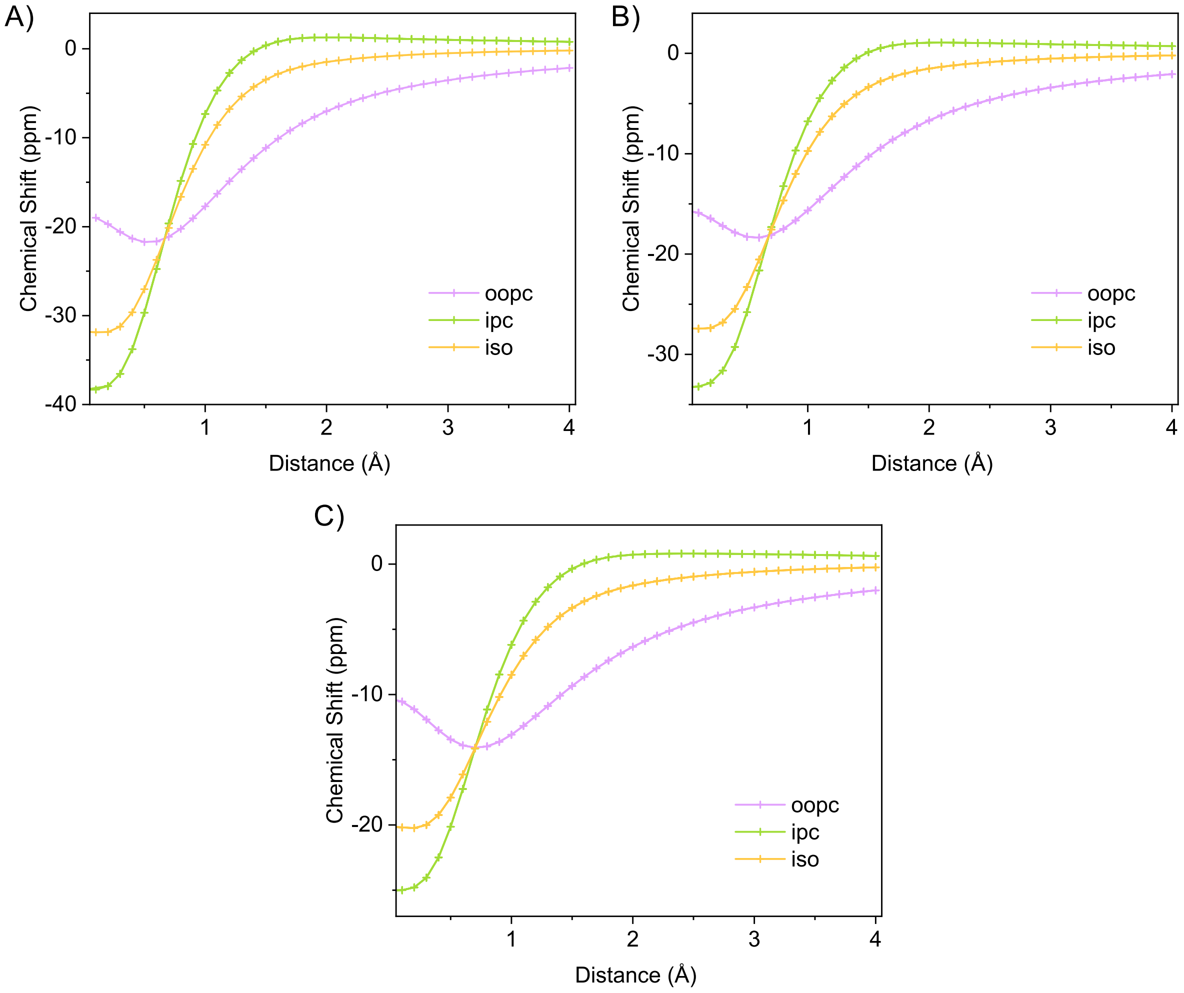

Figure S13: Calculated NICS values for **CN6CP**. The dianion **CN6CP^{2-}** (**A**), the radical anion **CN6CP$^{\bullet-}$** (**B**) and the neutral **CN6CP** (**C**). Depicted is the out-of-plane component (oopc, purple) which corresponds to the NICS$_{ZZ}$ values, the in-plane-components (green) which is defined as the average of the NICS$_{XX}$ and NICS$_{YY}$ and the isotropy (orange). The nucleus-independent chemical shift (NICS) values were scanned from the centre of the three-membered ring at the molecular plane (0 Å) to a distance of 4 Å. NICS values were calculated using the ωB97D3 level with the def2-TZVPD basis set. The NMR and NICS values were calculated with the conductor-like polarisable continuum model (CPCM) for DMSO (ε = 46.8 at 20 °C) as the solvation model.

The nucleus-independent chemical shift (NICS)[23] values were scanned from the centre of the three-membered ring in the molecular plane (0 Å) to a distance of up to 4 Å.[22] All three redox states show the overall similar behaviour. The out-of-plane component (oopc), exhibits a distinct minimum between -21.7 ppm@0.5 Å for the dianion **CN6CP^{2-}** which decreases to -14.1 ppm@0.7 Å for the neutral **CN6CP**. The in-plane component in the XY plane displays strongly negative NICS values that decrease with increasing distance and asymptotically approach zero. Overall, a gradual reduction of magnetic shielding is observed upon oxidation toward the electron-poorer redox states for both the out-of-plane and in-plane components. The isotropic chemical shift shows no local minimum around 1 Å, indicating the absence of aromaticity. Accordingly, the isotropic NICS values exhibit no minimum, confirming that the system is non-aromatic, as typically observed for cyclopropane systems.[24-26]

NICS Tabelle

Table S8: Calculated NICS(0), NICS(1) and NICS(1)$_{zz}$ values for all three oxidation states of **CN6CP**. Values were calculated at the ωB97D3 level with the def2-TZVPD basis set. The NMR and NICS values were calculated with the conductor-like polarizable continuum model (CPCM) for DMSO. A clear trend towards increasingly negative isotropic magnetic shielding is observed for the more electron-rich systems.

Molecule	NICS(0)	NICS(1)	NICS(1)$_{zz}$
Neutral (**CN6CP**)	-20.18	-8.48	-13.08
Radical-anion (**CN6CP**$^{\cdot-}$)	-27.40	-9.73	-15.64
Dianion (**CN6CP**$^{2-}$)	−31.33	−10.80	−17.84

HOMA Analysis for Cyclopropane Derivatives

The Harmonic Oscillator Model of Aromaticity (HOMA) is a geometric aromaticity index that is based exclusively on bond lengths (R_i). It quantifies how closely the bond lengths in a ring approach an optimal reference value (R_{opt}) associated with a fully delocalised system. Traditionally, benzene is used as the ideal aromatic reference compound and is assigned a HOMA value of 1. In Equation 2 n is the number of C-C bonds in the ring, and α (usually 257.7 Å^{-2}) normalizes the index to be unitless and equal to 0 for a hypothetical perfectly alternating Kekulé cyclohexatriene ring.

$$HOMA = 1 - \frac{\alpha}{n}\sum_{i=1}^{n}(R_{opt} - R_i)^2 \qquad \text{Eq. 2}$$

In the present work, this concept was adapted to three-membered rings. Owing to their unique bonding situation, which is commonly described by the Förster–Coulson–Moffitt model (“banana bonds”) or the Walsh model (often interpreted as “sp^5-like” hybridisation), C–C bonds in cyclopropane-derived systems are shorter than expected for typical single bonds. This led in our case to the paradox, that bond length shorter than 1.388 Å lead to lower HOMA values.[27] For this reason, the cyclopropenium cation was chosen as the ideal aromatic reference system for three-membered rings.

To the best of our knowledge, the parent cyclopropenium ion ($C_3H_3^+$) has only been isolated in the gas phase and no X-ray crystal structure has been reported. Therefore, a theoretically predicted C-C bond length of ~1.359 Å[28] was used as the reference value in our calculations. In contrast, experimentally determined X-ray structures of substituted cyclopropenium derivatives often show C-C bond lengths which mostly fall in the range of 1.37 to 1.38 Å.[29, 30] However, these derivatives usually contain electron-donating or electron-withdrawing substituents, bulky groups, or asymmetric substitution patterns that may influence the bond lengths. Since increased aromatic stabilisation is expected to result in shorter bond lengths, 1.359 Å was chosen as the reference value for an idealised aromatic three-membered ring.

Additionally, the empiric constant α is often set to 257.7 $Å^{-2}$ a valiue calculated from the optimal aromatic bond and a normal non aromatic σ-bond. However this value can be adapted to C3-rings.

$$\alpha_{C3} = \frac{1}{(R_{nonar} - R_{ar})^2} \qquad \text{Eq. 3}$$

As a non-aromatic system we choose cyclopropane C_3H_6 (1.501 Å) which can open to debate since it exhibit weak σ-aromaticity by itself.[31] However due to the unique bonding situations of Cyclopropane-derivates we found that it is a pragmatic localised reference. Resulting in an α_{C3} of 49.6 $Å^{-2}$.

The choice of the reference bond length significantly affects the resulting HOMA values. Consequently, the HOMA values reported here should not be directly compared with HOMA values derived for other aromatic systems.

Table S9: HOMA values calculated from the bond length determined by X-ray diffraction spectroscopy.[19] Two values are given one arose from Benzene as perfect aromatic system ($HOMA_{Benzen}$) and one from using cyclopropenium as a perfect aromatic system for C3-memberd cycles. Due to the unique bonding situation in C3-memberd cycles this HOMA values are not comparable to other HOMA values. The value marked with an asterix comes from bond length slightly shorter than benzene.

	Dianion	Radical anion	Neutral
$HOMA_{Benzen}$	0.996*	0.962	0.788
$HOMA_{C3}$	0.957	0.916	0.835

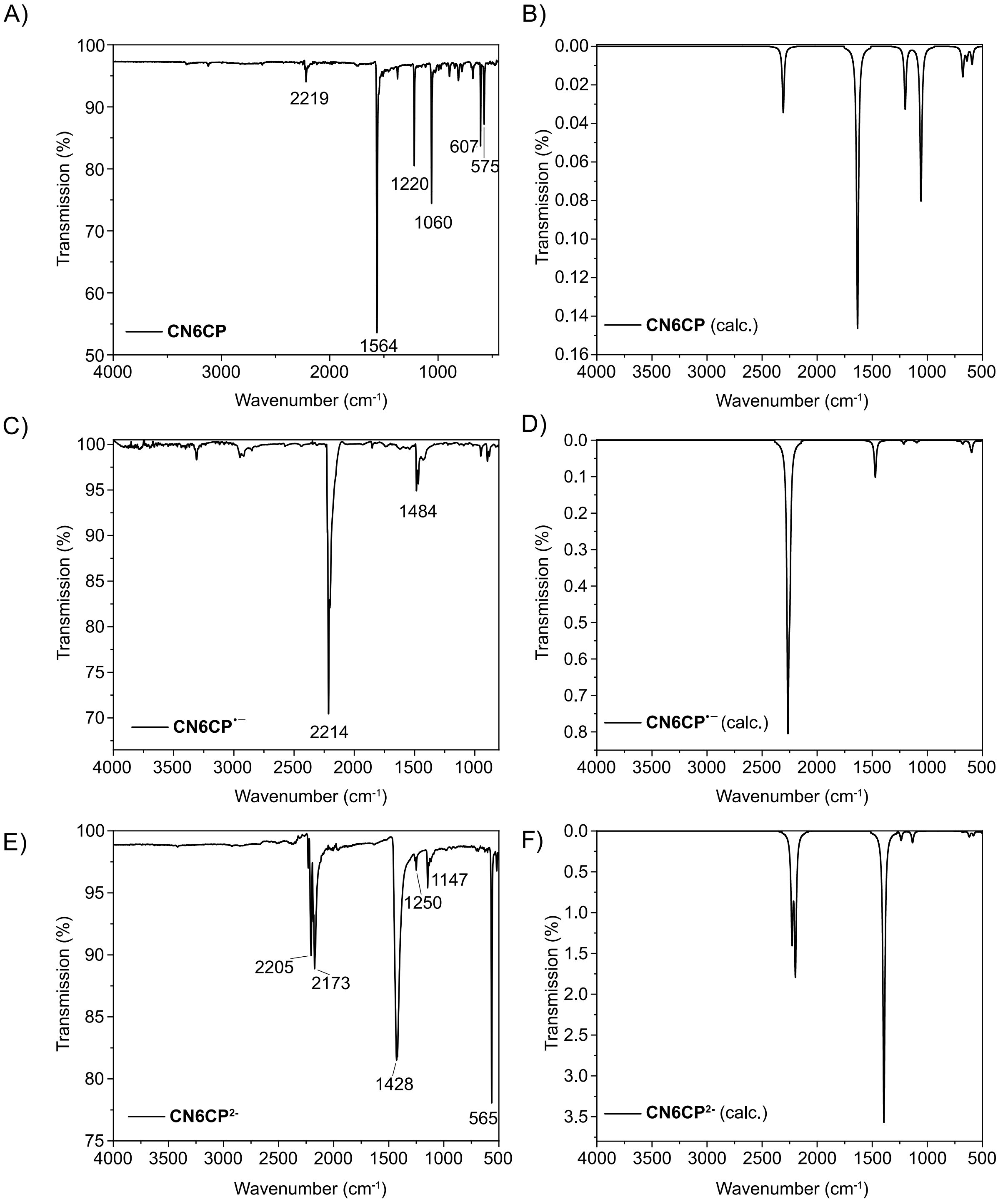

Figure S14: Experimental and calculated infrared (IR) spectra of all three oxidation states of **CN6CP**. **A)** experimental and **B)** calculated IR spectra of **CN6CP**; **C)** experimental IR spectra of **K^+[CN6CP]**$^{\cdot-}$and **D)** calculated IR spectra of **CN6CP**$^{\cdot-}$; **E)** experimental IR spectra of **Na^+_2[CN6CP]$^{2-}$** and **F)** calculated IR spectra of **CN6CP^{2-}**. Frequency calculations were performed at the ωB97X-D3/def2-TZVPD level, employing the RIJCOSX approximation with the def2/J auxiliary basis and a CPCM solvation model for acetonitrile.

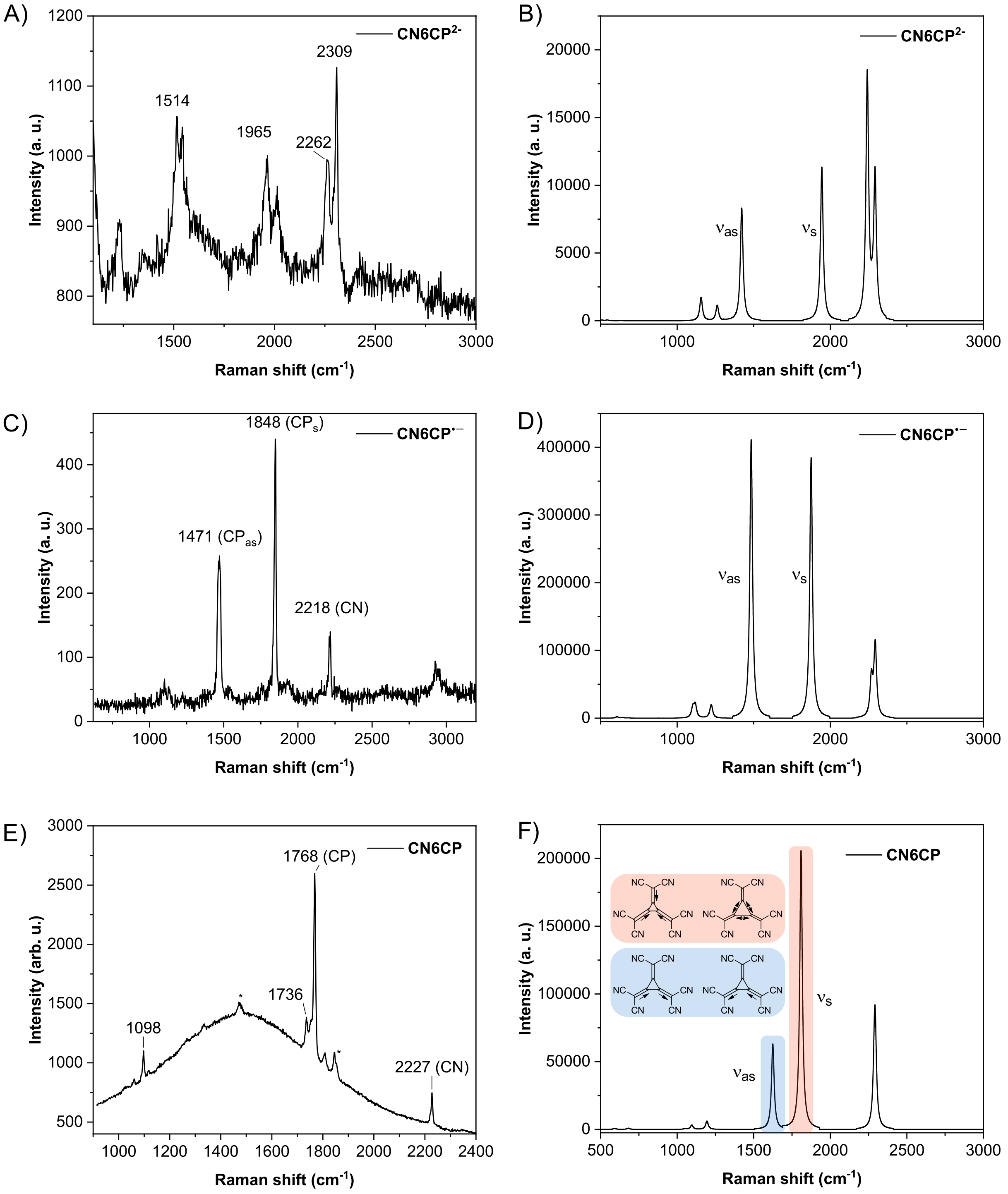

Figure S15: Measured and calculated Raman spectra of **CN6CP**$^{2-}$ (**A** and **B**) **CN6CP**$^{\cdot-}$ (**C** and **D**) und **CN6CP** (**E** and **F**). For **CN6CP** small amounts of **CN6CP**$^{\cdot-}$ are present, which are labelled with an asterisk. These impurities are most likely formed due to moisture in the air.

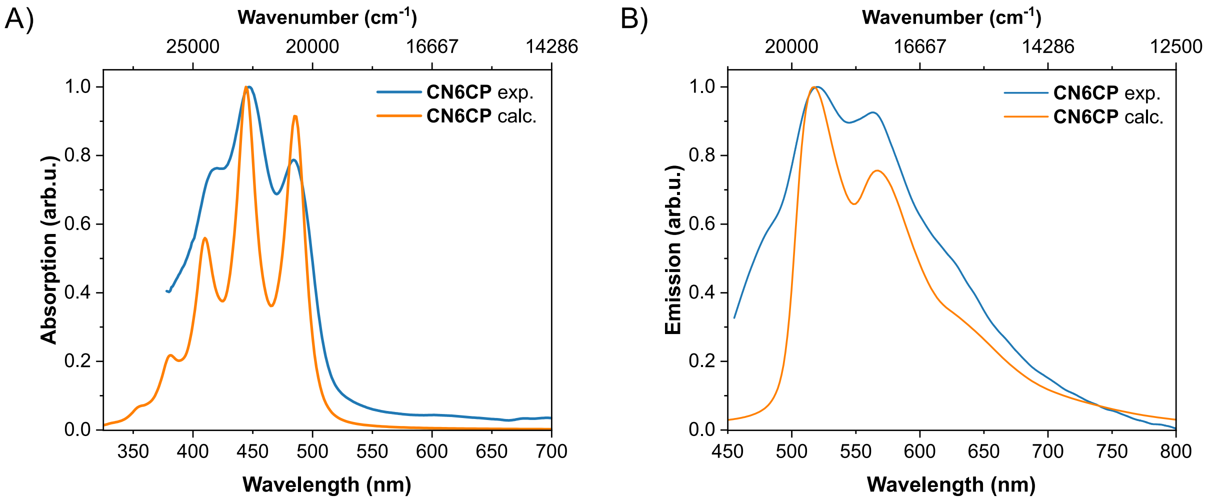

Figure S16: **A**) Experimental absorption spectra and **B**) emission spectra of CN6CP in MeCN/TFA (3:1) (blue) and the respective calculated spectra (orange). The spectra were calculated at the *ω*B97D3 level with the def2-TZVPD basis set. All values were obtained using the conductor-like polarizable continuum model (CPCM) for MeCN together with the ESD simulation implemented in ORCA 6.1. The calculated UV/Vis spectrum was rigidly shifted by a constant energy offset (ΔE = −0.21 eV) to align the band maxima with experiment; the vibronic structure and relative band shapes are taken directly from the ESD simulation. The distinct vibrational modes (see **Figure S14** and **S15**) dominated by the cyclopropane core account for the split absorption and emission bands, a phenomenon known as vibrational broadening, typical for dicyanomethylene -substituted [3]radialenes.

**Table S10**: TDDFT transitions of **CN6CP** calculate at the *ω*B97D3/def2-TZVPD level using CPCM(Acetonitrile). Overall, the UV/Vis spectra are red-shifted by approximatly −0.21 eV compared to the experimental spectra.
TD-DFT/TDA EXCITED STATES (SINGLETS)
Only dominant orbital contributions (>10%) are listed; minor configurations were omitted for clarity.
STATE 1: E= 0.099004 au 2.694 eV 21728.9 cm**-1 <S**2> = 0.000000 Mult 1
56a -> 57a : 0.973074 (c= 0.98644514)
STATE 2: E= 0.103520 au 2.817 eV 22719.9 cm**-1 <S**2> = 0.000000 Mult 1
55a -> 57a : 0.974603 (c= 0.98721971)
STATE 3: E= 0.159187 au 4.332 eV 34937.5 cm**-1 <S**2> = 0.000000 Mult 1
54a -> 57a : 0.787488 (c= 0.88740546)
STATE 4: E= 0.163947 au 4.461 eV 35982.2 cm**-1 <S**2> = 0.000000 Mult 1
52a -> 57a : 0.660079 (c= 0.81245261)
53a -> 57a : 0.204966 (c= -0.45273197)
STATE 5: E= 0.170658 au 4.644 eV 37455.0 cm**-1 <S**2> = 0.000000 Mult 1
52a -> 57a : 0.194801 (c= 0.44136234)
53a -> 57a : 0.616501 (c= 0.78517571)
STATE 6: E= 0.182742 au 4.973 eV 40107.2 cm**-1 <S**2> = 0.000000 Mult 1
47a -> 57a : 0.825972 (c= 0.90882980)
STATE 7: E= 0.183125 au 4.983 eV 40191.3 cm**-1 <S**2> = 0.000000 Mult 1
48a -> 57a : 0.821296 (c= 0.90625390)
STATE 8: E= 0.189818 au 5.165 eV 41660.1 cm**-1 <S**2> = 0.000000 Mult 1
46a -> 57a : 0.883211 (c= 0.93979328)
STATE 9: E= 0.195855 au 5.329 eV 42985.3 cm**-1 <S**2> = 0.000000 Mult 1
51a -> 57a : 0.848331 (c= 0.92104872)
STATE 10: E= 0.196255 au 5.340 eV 43073.1 cm**-1 <S**2> = 0.000000 Mult 1
50a -> 57a : 0.849408 (c= 0.92163334)

Scanning Tunneling microscopy (STM)

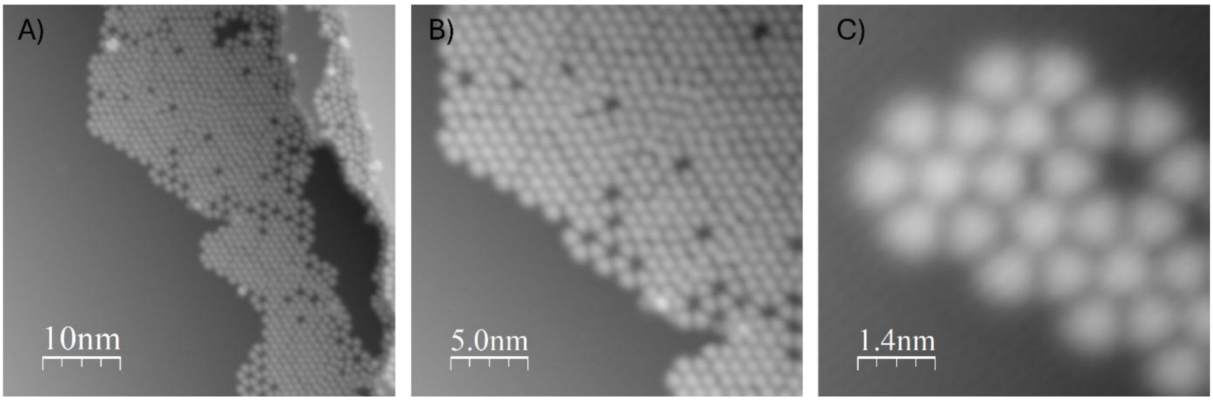

Figure S17: Scanning tunnelling microscopy (STM) images of **A**), **B**) and **C**) show **CN6CP** evaporated at 170 °C under ultra-high vacuum onto a Cu(111) surface held at room temperature. The molecules are densely packed and flat on the surface. A trigonal (honeycomb) arrangement for the molecules is observed. **B**) is a close-up view of (**A**). The images were recorded in constant-current mode with I = 50 pA, U = 1.0 V.

The flat-lying adsorption geometry is consistent with favourable molecule surface interactions of the largely planar **CN6CP** framework on Cu(111). However, the relatively close-packed arrangement, despite the presence of neighbouring cyano groups, suggests that additional substrate-mediated stabilisation is operative. In line with previous studies on cyano-functionalised molecules on metal surfaces, Cu adatoms may act as coordination sites and thereby contribute to the observed surface assembly. The STM results further demonstrate that neutral **CN6CP** can be sublimed intact under ultra-high vacuum conditions and deposited on Cu(111) while preserving its molecular integrity.

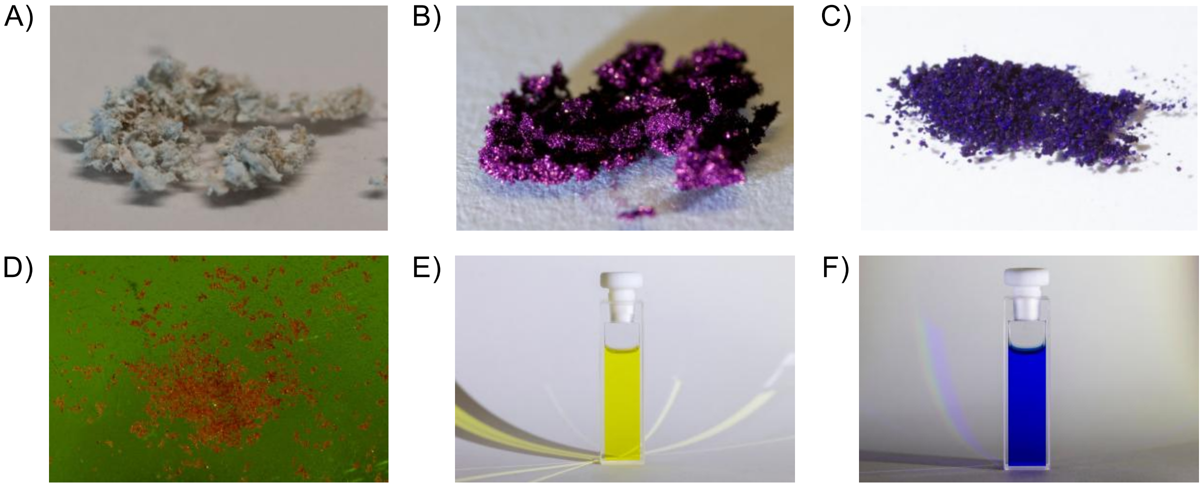

Figure S18: Photos of solid **A) Na$^{+}_{2}$[CN6CP]$^{2-}$**, **B) K^{+}[CN6CP]$^{\cdot-}$** and **C) NBu$_4^{+}$[CN6CP]$^{\cdot-}$**. **D)** solid neutral **CN6CP** crystallised from a solution of MeCN/TFA (3:1) with s small amount of NOSbF$_6$. **E)** Dissolved neutral **CN6CP** in MeCN/TFA (3:1) and **F)** dissolved radical anion **K^{+}[CN6CP]$^{\cdot-}$** in MeCN.

Single Crystal X-Ray Crystallographic (XRD)

**Table S11:** Crystal data and structure refinement details for the radical anion **K^+[CN6CP]$^{\cdot-}$**.The same cell parameter for this crystal have been reported previously.[32]	
Identification code	1875060
Empirical formula	$C_{12}KN_6$
Formula weight	267.272
Temperature [K]	150(2)
Crystal system	triclinic
Space group	P-1 (1)
a [Å]	6.5731(2)
b [Å]	8.7746(2)
c [Å]	11.1018(4)
a [°]	104.486(1)
β [°]	94.177(1)
γ [°]	107.178(1)
Volume [$Å^3$]	584.80(3)
Z	2
ρ_{calc} [g/cm^3]	1.518
μ/mm^{-1}	0.447
F(000)	266.6
Crystal size [mm^3]	0.37 × 0.28 × 0.09
Radiation	MoKα (λ = 0.71073 Å)
2θ range [°]	5.06 to 52.84
Index ranges	-8 ≤ h ≤ 8<br>-10 ≤ k ≤ 10<br>-13 ≤ l ≤ 13
Reflections collected	12039
Independent reflections	2371<br>R_{int} = 0.0597<br>R_{sigma} = 0.0387
Data/restraints/parameters	2371/0/172
Goodness-of-fit on F^2	1.039
Final R indexes [I>=2σ (I)]	R_1 = 0.0318<br>wR_2 = 0.0749
Final R indexes [all data]	R_1 = 0.0353<br>wR_2 = 0.0783
Largest diff. peak/hole / e $Å^{-3}$	0.25/-0.22

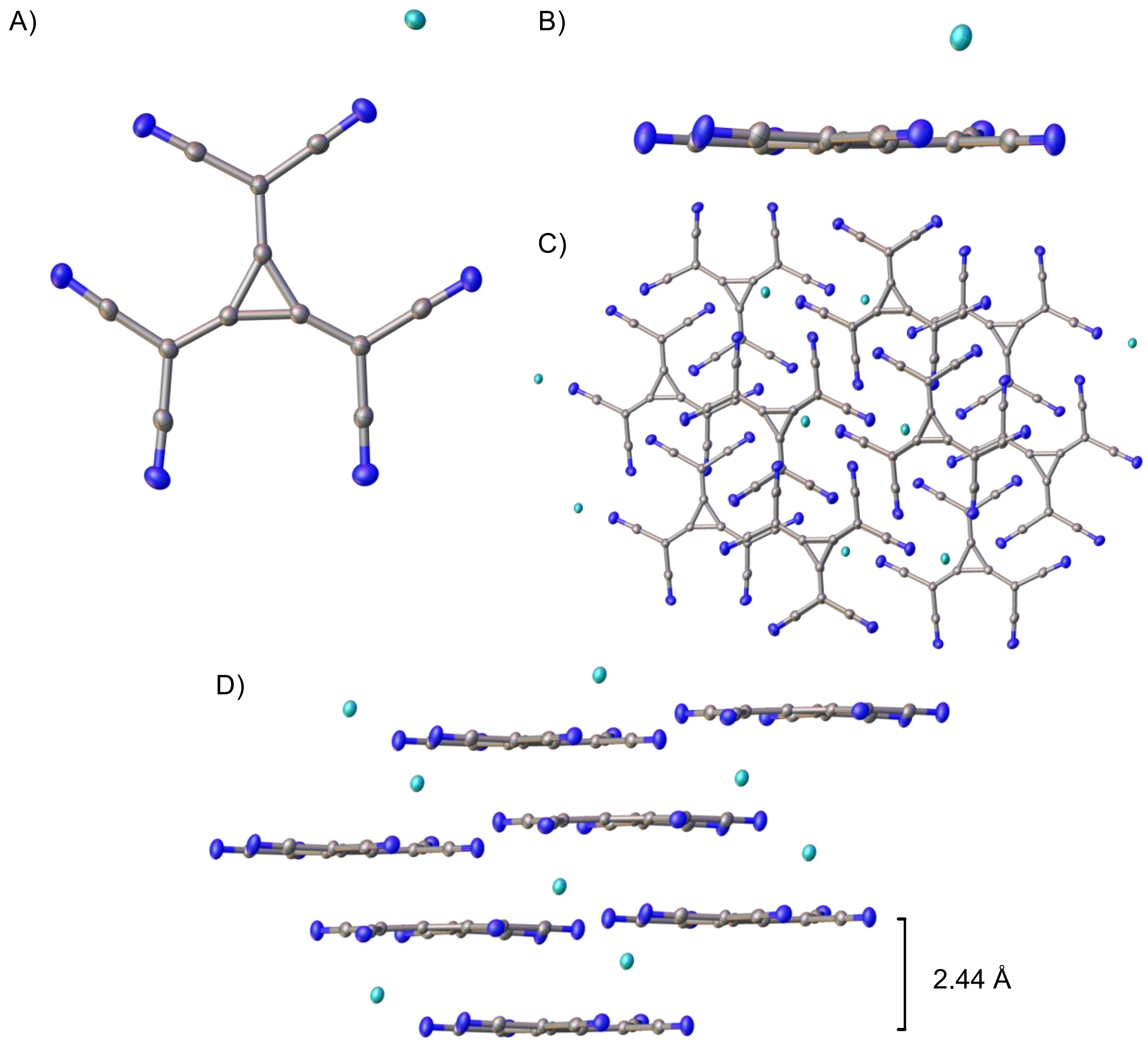

Figure S19: Single-crystal X-ray structure of **K⁺[CN6CP]·⁻**. **A)** front view and **B)** side view of the structure of **K⁺[CN6CP]·⁻**. **C)** Crystal packing and **D)** crystal stacking of **K⁺[CN6CP]·⁻**. Displacement ellipsoids are drawn at the 50% probability level.

**Table S12:** Crystal data and structure refinement details for **CN6CP**.	
CCDC number	2496539
Empirical formula	$C_{14}H_3N_7$
Formula weight	269.23
Temperature [K]	167(2)
Crystal system	orthorhombic
Space group (number)	$Pnma$ (62)
a [Å]	9.4267(12)
b [Å]	11.8644(13)
c [Å]	12.0518(16)
α [°]	90
β [°]	90
γ [°]	90
Volume [Å^3]	1347.9(3)
Z	4
ρ_{calc} [gcm^{-3}]	1.327
μ [mm^{-1}]	0.089
F(000)	544
Crystal size [mm^3]	0.120×0.133×0.142
Crystal colour	orange
Crystal shape	prism
Radiation	MoK_α (λ=0.71073 Å)
2θ range [°]	4.82 to 56.67 (0.75 Å)
Index ranges	−12 ≤ h ≤ 12<br>−15 ≤ k ≤ 15<br>−16 ≤ l ≤ 16
Reflections collected	21373
Independent reflections	1755<br>R_{int} = 0.0779<br>R_{sigma} = 0.0323
Completeness to θ = 25.242°	99.9 %
Data / Restraints / Parameters	1755 / 0 / 105
Absorption correction T_{min}/T_{max}	0.6993 / 0.7457(multi-scan)
Goodness-of-fit on F^2	1.061
Final *R* indexes [I≥2σ(I)]	R_1 = 0.0384<br>wR_2 = 0.0941
Final *R* indexes<br>[all data]	R_1 = 0.0564<br>wR_2 = 0.1064
Largest peak/hole [eÅ^{-3}]	0.24/−0.18
Extinction coefficient	0.0084(15)

Singel crystals were obtained from recrystallization of **CN6CP** from MeCN/TFA (4:1) with a small amount of $NOSbF_6$ dissolved. The crystals formed over 10 d at −25°C. A orange, prism-shaped crystal was measured at 167(2) K Crystallographic data for the structures reported in this paper have been deposited with the Cambridge Crystallographic Data Centre. CCDC 2496539[33] contain the supplementary crystallographic data for this paper. These data can be obtained free of charge from the Cambridge Crystallographic Data Centre via www.ccdc.cam.ac.uk/structures.

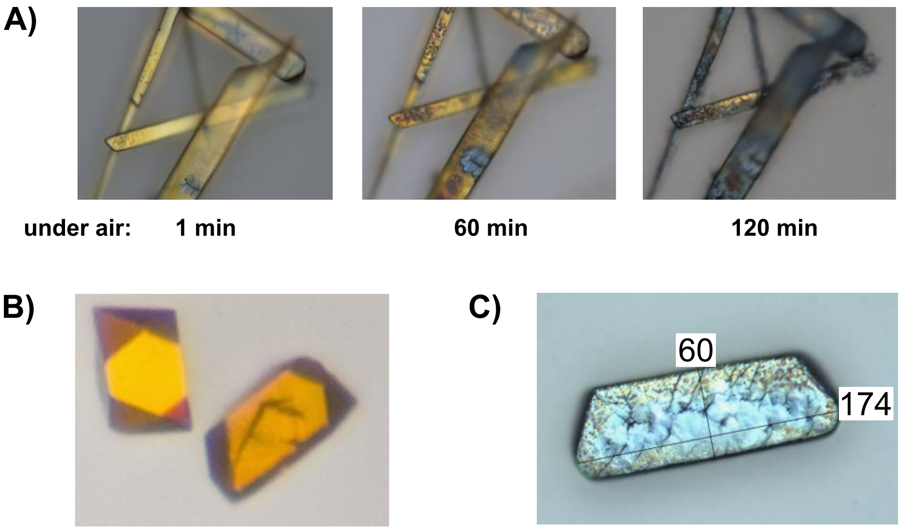

Figure S20: Crystals of **CN6CP**. **A)** A gradual colour change is observed over time upon exposure to **(moist)** air, most likely due to formation of the radical anion. **B)** Photographs of **CN6CP** crystals under an inert atmosphere. **C)** Example of a crystal distances are given in µm.

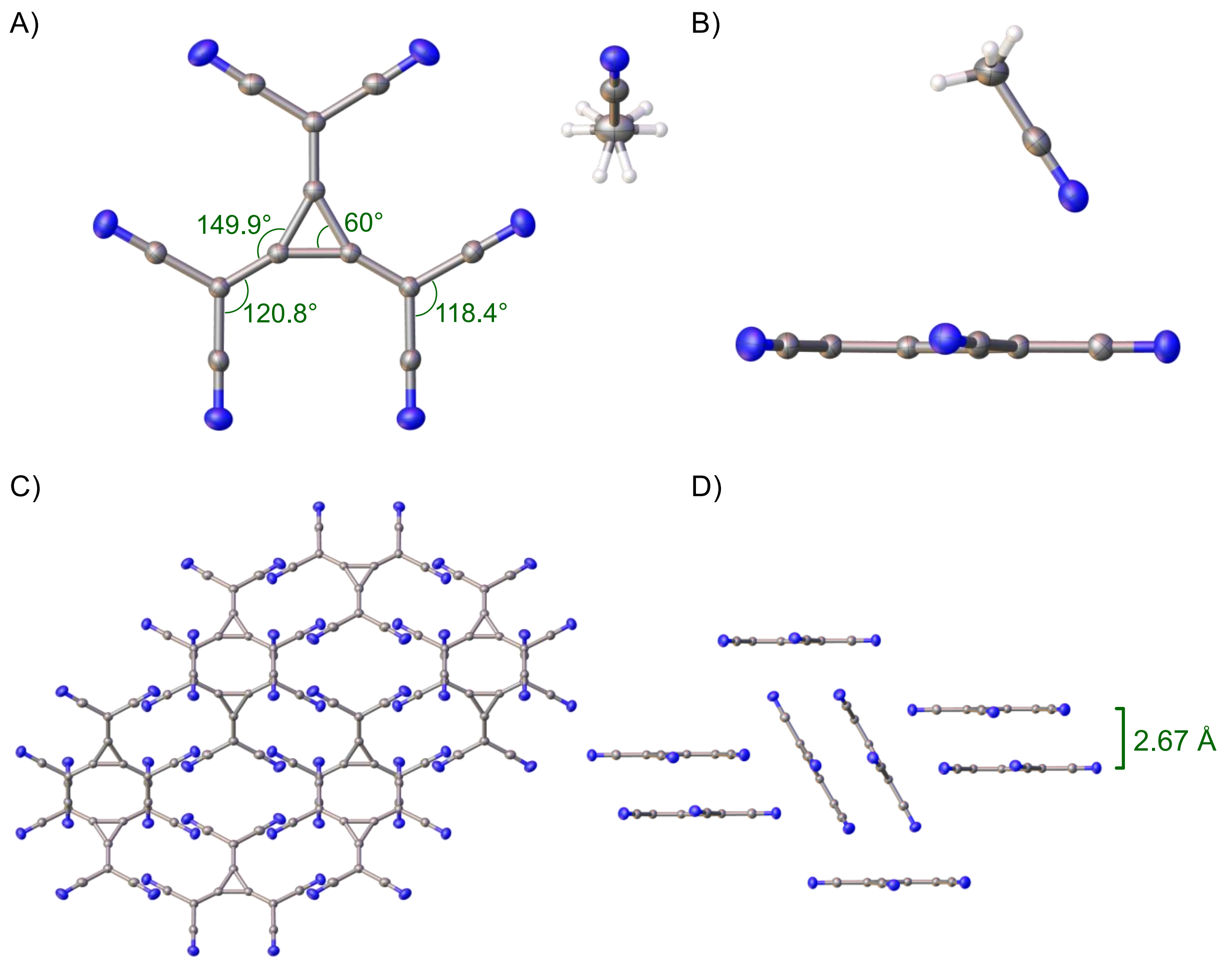

Figure S21: Single-crystal X-ray structure of **CN6CP**. **A)** Front view with specified bond angles and **B)** side view of the structure of **CN6CP**. **C)** Crystal packing and **D)** stacking of **CN6CP**. The molecule crystallizes with one equivalent of MeCN, which is not shown in panels **C**, and **D** for clarity. Displacement ellipsoids are drawn at the 50% probability level.

Single-crystal X-ray diffraction shows that **CN6CP** exhibits crystallographic C2 symmetry; however, the deviations from idealised C3 symmetry are within experimental uncertainty. The molecule is essentially planar. The dicyanomethylene substituents are only slightly twisted with respect to the plane of the cyclopropane core by 3.12(8)° and 1.51(13)°, respectively. The C–C bond lengths within the cyclopropane ring range from 1.4152(17) to 1.4174(18) Å, and the internal bond angles are close to the ideal value of 60°. The exocyclic C–C bonds connecting the ring to the dicyanomethylene units range from 1.3495(17) to 1.3502(24) Å. The bond lengths between the central carbon atoms of the dicyanomethylene groups and the nitrile carbon atoms lie between 1.4323(17) and 1.4367(16) Å in neutral **CN6CP**.

In the crystal, **CN6CP** adopts a sandwich-herringbone-like packing motif, which is most clearly seen in projection along the (010) plane. The structure consists of slipped antiparallel rows of **CN6CP** molecules that are offset with respect to one another by

approximately half a molecular length. One equivalent of lattice MeCN is co-crystallised and occupies an interstitial site between adjacent **CN6CP** molecules.

A notable feature of the packing is the prevalence of short intermolecular N to C contacts. The nitrile nitrogen atoms repeatedly approach carbon atoms of neighbouring **CN6CP** molecules, as well as the lattice MeCN, at distances at or slightly below the sum of the van der Waals radii, indicating close intermolecular contacts. These include contacts between antiparallel **CN6CP** molecules as well as approximately perpendicular contacts between neighbouring rows. The shortest MeCN to **CN6CP** contact is observed at approximately 3.172 Å. Within the **CN6CP** packing, additional short N to C contacts in the range of approximately 3.19-3.33 Å are observed, consistent with a network of weak but recurrent intermolecular interactions. Very weak C-H to N contacts involving the lattice MeCN may further contribute to the overall packing, but the dominant motif is the dense network of short N to C contacts.

Compared with neutral **CN6CP**, the crystallographically characterised radical anion **K^+[CN6CP]$^{\cdot-}$**[32] and dianion **$(NBu_4)^+_2$[CN6CP]$^{2-}$**[19] show systematic, although individually small, changes in bond lengths upon reduction. Most notably, the C−C bonds within the cyclopropane ring become shorter, decreasing from 1.4152(17)-1.4174(18) Å in neutral **CN6CP** to 1.385(5)-1.393(7) Å in **$(NBu_4)^+_2$[CN6CP]$^{2-}$**. By contrast, the exocyclic C−C bonds connecting the ring to the dicyanomethylene units become longer, increasing from 1.3495(17)-1.3502(24) Å in neutral **CN6CP** to 1.386(7)-1.407(5) Å in the dianion. At the same time, the bonds between the central carbon atoms of the dicyanomethylene groups and the nitrile carbon atoms shorten from 1.4323(17)-1.4367(16) Å in neutral **CN6CP** to 1.411(9)-1.421(8) Å in the dianion. Although some individual differences within each structure are close to the experimental uncertainty, the overall trend across the neutral species, radical anion, and dianion is consistent with progressive shortening of the ring C−C bonds and concomitant lengthening of the exocyclic C−C bonds upon reduction. In the overall packing of the radical anion is dominated by coordination of the Nitrile N-atoms to the K^+ cations. No CN−CN dipolar interactions can be observed.

Electron paramagnetic resonance (EPR)

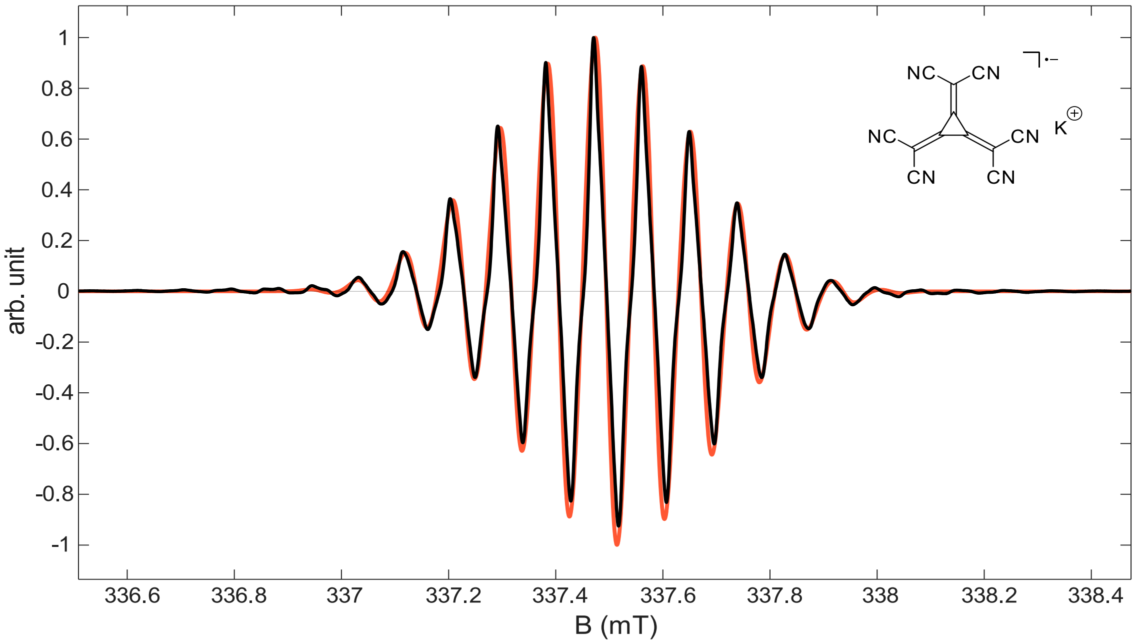

Figure S22: Electron paramagnetic resonance (EPR) spectra of the radical anion **K^+[CN6CP]$^{\cdot-}$**. X-band CW-EPR spectrum recorded at 295 K (ν = 9.46 GHz, B = 335–340 mT, modulation = 0.05 mT @ 100 kHz, P = 10 mW, sweep = 60 s). The experimental spectrum (black) is shown together with a simulated spectrum (red) using EasySpin[3] (g_{iso} = 2.00267, A(^{14}N)=2.48 MHz, ΔB_{pp} = 0.042 mT) with a root mean square deviation (RMSD) of 0.043.

The X-band CW-EPR spectrum of the radical anion **CN6CP**$^{\cdot-}$ recorded at 295 K (ν = 9.46 GHz) shows a slightly broadened single line signal with weak superhyperfine structure arising from coupling to nitrogen nuclei (**Figure 22**). Simulation using EasySpin reproduces the experimental line shape using g_{iso} = 2.00296, a linewidth of ΔB_{pp} = 0.042 mT, and an isotropic hyperfine coupling of A(^{14}N) = 2.48 MHz.

Density functional theory (DFT) calculations performed at the TPSSh/EPR-III and using the conductor-like polarizable continuum model CPCM(Acetonitrile) predict A_{iso}(^{14}N) ≈ 2.3 MHz, in good agreement with the experiment. The small magnitude of the ^{14}N hyperfine coupling relative to the linewidth explains why no fully resolved nitrogen triplets are visible and why the spectrum appears as a broadened single line. The experimental and computed spin distributions consistently show that the unpaired electron is delocalized mainly delocalised over the dicyanomethylene substituents, with only minor spin density on the cyclopropane ring atoms. Analysis of the calculated A_{iso}-values clearly indicates that the unpaired electron resides predominantly on the dicyanomethylene carbon atoms, with a smaller but significant contribution on the nitrile nitrogen atoms. This is fully consistent with the computed spin-density distribution (**Table S3**: (Mulliken) spin populations; **Figure S7**: spin-density isosurface), which shows the same trend for different functionals and basis sets: the major spin population is located on the dicyanomethylene carbons and on the nitrogen atoms, whereas the cyclopropane carbon atoms carry only very small, partly negative spin populations arising from σ-spin polarisation. Together, these observations unambiguously confirm that the radical is predominantly delocalized within the exocyclic dicyanomethylene π-system and only to a minor extent on the cyclopropane ring.

Nuclear magnetic resonance spectroscopy (NMR)

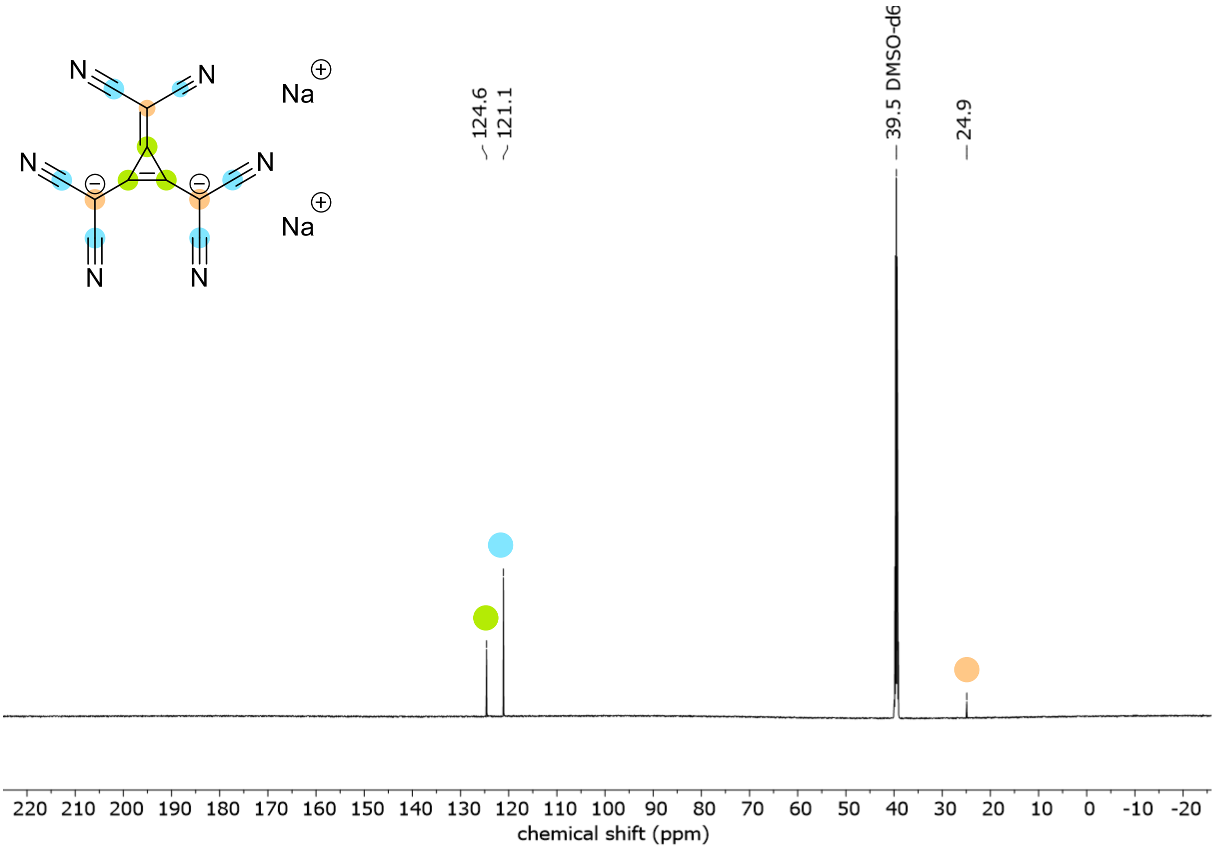

Figure S23: ^{13}C NMR (151 MHz, DMSO-d_6) of disodium 2,2',2''-(cyclopropane-1,2,3-triylidene)trimalononitrile.

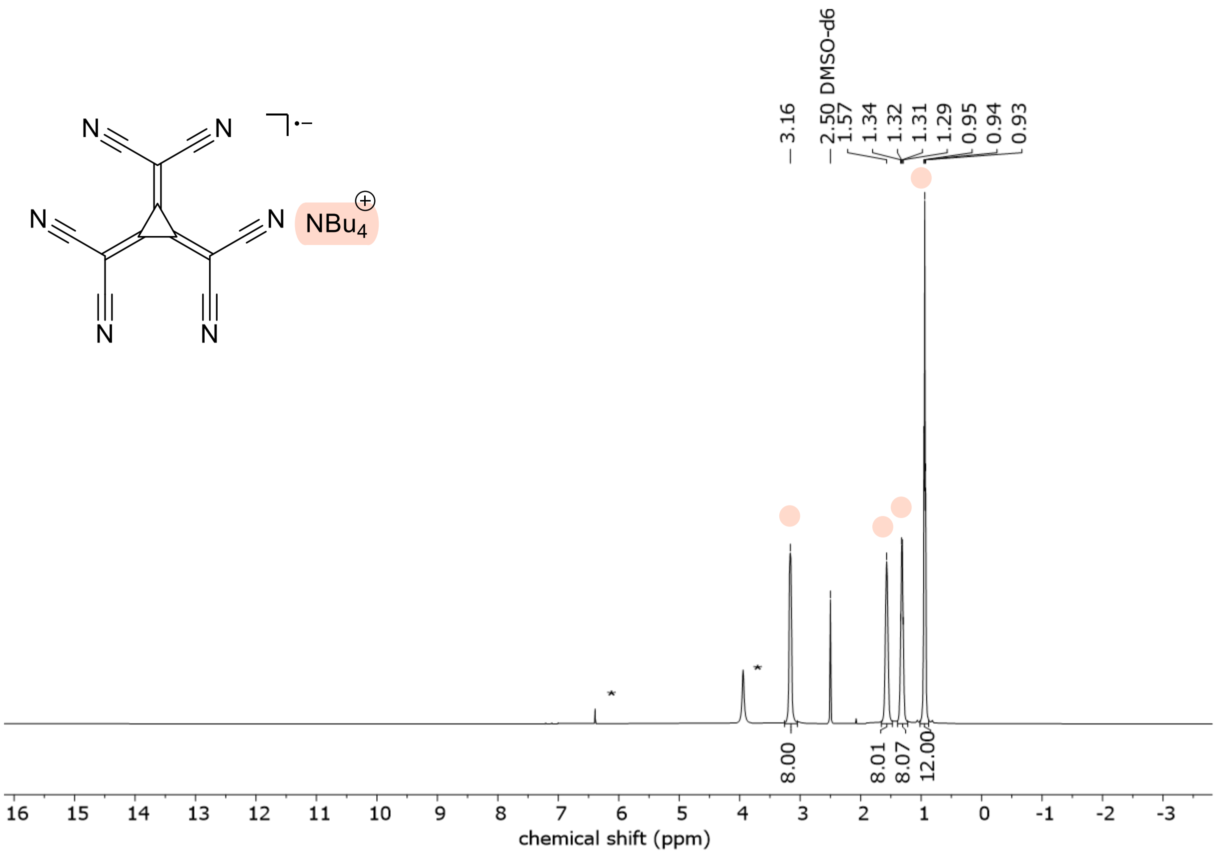

Figure S24: ^{1}H NMR (500 MHz, DMSO-d_6) tetrabutylammonium 2,2',2''-(cyclopropane-1,2,3-triylidene)trimalononitrile anion radical

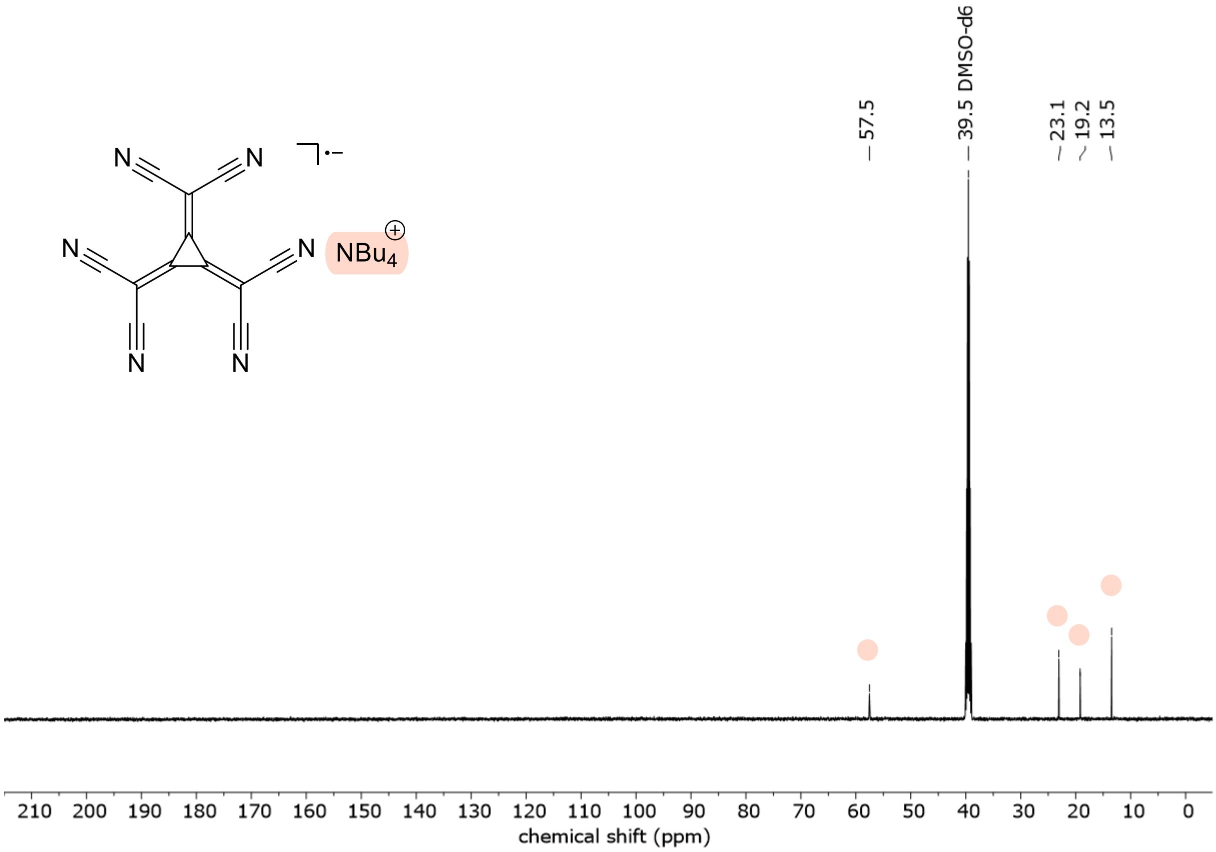

Figure S25: ^{13}C NMR (126 MHz, DMSO-d_6) of tetrabutylammonium 2,2',2''-(cyclopropane-1,2,3-triylidene)trimalononitrile anion radical.

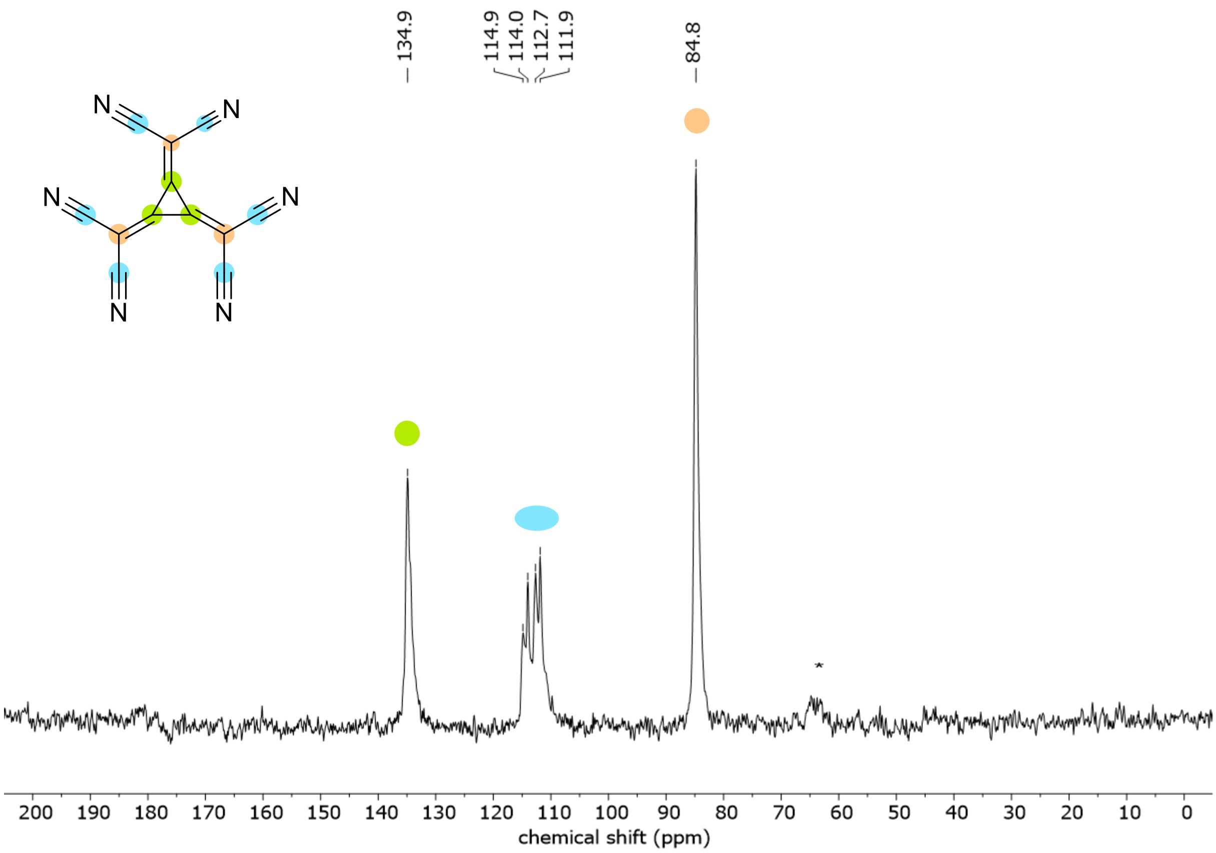

Figure S26: Solid-state ^{13}C NMR (300 MHz) of 2,2',2''-(cyclopropane-1,2,3-triylidene)trimalononitrile. The signal at 64.8 ppm, marked with an asterisk, corresponds to residual MeCN present in the crystals, which is difficult to remove completely without inducing decomposition of the compound. Consequently, a minor contribution to the signal in the range from 114.9 to 111.9 ppm is also attributed to acetonitrile.

Referances

[1] M. R. Willcott, MestRe Nova, *J. Am. Chem. Soc.* **2009**, *131*, 13180–13180.

[2] G. R. Fulmer, A. J. M. Miller, N. H. Sherden, H. E. Gottlieb, A. Nudelman, B. M. Stoltz, J. E. Bercaw, K. I. Goldberg, NMR Chemical Shifts of Trace Impurities: Common Laboratory Solvents, Organics, and Gases in Deuterated Solvents Relevant to the Organometallic Chemist, *Organometallics* **2010**, *29*, 2176–2179.

[3] S. Stoll, A. Schweiger, EasySpin, a comprehensive software package for spectral simulation and analysis in EPR, *J. Magn. Reson.* **2006**, *178*, 42-55.

[4] C. Würth, M. Grabolle, J. Pauli, M. Spieles, U. Resch-Genger, Relative and absolute determination of fluorescence quantum yields of transparent samples, *Nat. Protoc.* **2013**, *8*, 1535–1550.

[5] G. Sheldrick, A short history of SHELX, *Acta Crystallogr., Sect. A* **2008**, *64*, 112–122.

[6] N. Elgrishi, K. J. Rountree, B. D. McCarthy, E. S. Rountree, T. T. Eisenhart, J. L. Dempsey, A Practical Beginner's Guide to Cyclic Voltammetry, *J. Chem. Educ.* **2018**, *95*, 197-206.

[7] F. Neese, Software Update: The ORCA Program System—Version 6.0, *Comput. Mol. Sci.* **2025**, *15*, e70019.

[8] F. Neese, The ORCA program system, *Comput. Mol. Sci.* **2012**, *2*, 73-78.
[9] B. P. Pritchard, D. Altarawy, B. Didier, T. D. Gibson, T. L. Windus, New Basis Set Exchange: An Open, Up-to-Date Resource for the Molecular Sciences Community, *J. Chem. Inf. Model.* **2019**, *59*, 4814-4820.
[10] Y.-S. Lin, G.-D. Li, S.-P. Mao, J.-D. Chai, Long-Range Corrected Hybrid Density Functionals with Improved Dispersion Corrections, *J. Chem. Theory Comput.* **2013**, *9*, 263-272.
[11] I. M. Smallwood, in *Handbook of Organic Solvent Properties*, Elsevier Ltd., Oxford, **1996**.
[12] V. Barone, M. Cossi, Quantum Calculation of Molecular Energies and Energy Gradients in Solution by a Conductor Solvent Model, *J. Phys. Chem. A* **1998**, *102*, 1995-2001.
[13] D. Mester, M. Kállay, Charge-Transfer Excitations within Density Functional Theory: How Accurate Are the Most Recommended Approaches?, *J. Chem. Theory Comput.* **2022**, *18*, 1646-1662.
[14] M. J. Frisch, H. B. S. G. W. Trucks, G. E. Scuseria, M. A. Robb, J. R. Cheeseman, G., V. B. Scalmani, G. A. Petersson, H. Nakatsuji, X. Li, M. Caricato, A. V. Marenich,, B. G. J. J. Bloino, R. Gomperts, B. Mennucci, H. P. Hratchian, J. V. Ortiz, A. F., J. L. S. Izmaylov, Williams, F. Ding, F. Lipparini, F. Egidi, J. Goings, B. Peng,, T. H. A. Petrone, D. Ranasinghe, V. G. Zakrzewski, J. Gao, N. Rega, G., W. L. Zheng, M. Hada, M. Ehara, K. Toyota, R. Fukuda, J. Hasegawa, M. Ishida,, Y. H. T. Nakajima, O. Kitao, H. Nakai, T. Vreven, K. Throssell, J. A. Montgomery, J. E. P. Jr., F. Ogliaro, M. J. Bearpark, J. J. Heyd, E. N. Brothers, K. N. Kudin, V., T. A. K. N. Staroverov, R. Kobayashi, J. Normand, K. Raghavachari, A. P. Rendell,, S. S. I. J. C. Burant, J. Tomasi, M. Cossi, J. M. Millam, M. Klene, C. Adamo, R., J. W. O. Cammi, R. L. Martin, K. Morokuma, O. Farkas, J. B. Foresman, D. J., *Fox, Wallingford, CT,* **2016.**
[15] T. Lu, F. Chen, Multiwfn: A multifunctional wavefunction analyzer, *J. Comput. Chem.* **2012**, *33*, 580-592.
[16] T. Lu, A comprehensive electron wavefunction analysis toolbox for chemists, Multiwfn, *J. Chem. Phys.* **2024**, *161*.
[17] E. F. Pettersen, T. D. Goddard, C. C. Huang, G. S. Couch, D. M. Greenblatt, E. C. Meng, T. E. Ferrin, UCSF Chimera—A visualization system for exploratory research and analysis, *J. Comput. Chem.* **2004**, *25*, 1605-1612.
[18] D. W. Szczepanik, M. Andrzejak, J. Dominikowska, B. Pawełek, T. M. Krygowski, H. Szatylowicz, M. Solà, The electron density of delocalized bonds (EDDB) applied for quantifying aromaticity, *Phys. Chem. Chem. Phys.* **2017**, *19*, 28970-28981.
[19] J. Park, A. M. Houser, S. Zhang, A High-Voltage n-type Organic Cathode Materials Enabled by Tetraalkylammonium Complexing Agents for Aqueous Zinc-Ion Batteries, *Adv. Battery Mater.* **2024**, *36*, 2409946.
[20] J. Contreras-García, E. R. Johnson, S. Keinan, R. Chaudret, J.-P. Piquemal, D. N. Beratan, W. Yang, NCIPLOT: A Program for Plotting Noncovalent Interaction Regions, *Journal of Chemical Theory and Computation* **2011**, *7*, 625-632.
[21] E. R. Johnson, S. Keinan, P. Mori-Sánchez, J. Contreras-García, A. J. Cohen, W. Yang, Revealing Noncovalent Interactions, *J. Am. Chem. Soc.* **2010**, *132*, 6498-6506.
[22] Z. Wang, py.Aroma: An Intuitive Graphical User Interface for Diverse Aromaticity Analyses, *Chemistry* **2024**, *6*, 1692-1703.

[23] P. v. R. Schleyer, C. Maerker, A. Dransfeld, H. Jiao, N. J. R. van Eikema Hommes, Nucleus-Independent Chemical Shifts: A Simple and Efficient Aromaticity Probe, *J. Am. Chem. Soc.* **1996**, *118*, 6317-6318.

[24] A. Stanger, NICS – Past and Present, *Eur. J. Org. Chem.* **2020**, *2020*, 3120–3127.

[25] A. Stanger, Nucleus-Independent Chemical Shifts (NICS): Distance Dependence and Revised Criteria for Aromaticity and Antiaromaticity, *J. Org. Chem.* **2006**, *71*, 883–893.

[26] A. Stanger, Obtaining Relative Induced Ring Currents Quantitatively from NICS, *J. Org. Chem.* **2010**, *75*, 2281–2288.

[27] S. Ostrowski, J. C. Dobrowolski, What does the HOMA index really measure?, *RSC Adv.* **2014**, *4*, 44158-44161.

[28] X. Huang, P. R. Taylor, T. J. Lee, Highly Accurate Quartic Force Fields, Vibrational Frequencies, and Spectroscopic Constants for Cyclic and Linear C3H3+, *J. Phys. Chem. A* **2011**, *115*, 5005-5016.

[29] A. de Meijere, D. Faber, M. Noltemeyer, R. Boese, T. Haumann, T. Müller, M. Bendikov, E. Matzner, Y. Apeloig, Tris(trimethylsilyl)cyclopropenylium Cation: The First X-ray Structure Analysis of an α-Silyl-Substituted Carbocation, *J. Org. Chem.* **1996**, *61*, 8564-8568.

[30] K. Komatsu, T. Kitagawa, Cyclopropenylium Cations, Cyclopropenones, and HeteroanaloguesRecent Advances, *Chem. Rev.* **2003**, *103*, 1371-1428.

[31] W. Wu, B. Ma, J. I-Chia Wu, P. v. R. Schleyer, Y. Mo, Is Cyclopropane Really the σ-Aromatic Paradigm?, *Chem.–Eur. J.* **2009**, *15*, 9730-9736.

[32] H. Phan, T. S. Herng, D. Wang, X. Li, W. Zeng, J. Ding, K. P. Loh, A. T. Shen Wee, J. Wu, Room-Temperature Magnets Based on 1,3,5-Triazine-Linked Porous Organic Radical Frameworks, *Chem* **2019**, *5*, 1223-1234.

[33] Deposition numbers 2496539 (for **CN6CP**), 1875060 (for **K^+[CN6CP]**$^{\cdot -}$) and 2366307 (for **$(NBu_4)^{+2}$[CN6CP]**$^{2-}$) contain the supplementary crystallographic data for this paper, *these Data are Provided Free of Charge by the Joint Cambridge Crystallographic Data Centre ad Fachinformationszentrum Karlsruhe Access Structures Service.*
